# Carbonyl reductase 1 amplifies glucocorticoid action in adipose tissue and impairs glucose tolerance in lean mice

**DOI:** 10.1016/j.molmet.2021.101225

**Published:** 2021-03-27

**Authors:** Rachel M.B. Bell, Elisa Villalobos, Mark Nixon, Allende Miguelez-Crespo, Lee Murphy, Angie Fawkes, Audrey Coutts, Matthew G.F. Sharp, Martha V. Koerner, Emma Allan, Onno C. Meijer, Renè Houtman, Alex Odermatt, Katharina R. Beck, Scott G. Denham, Patricia Lee, Natalie Z.M. Homer, Brian R. Walker, Ruth A. Morgan

**Affiliations:** 1 British Heart Foundation Centre for Cardiovascular Science, The Queen's Medical Research Institute, University of Edinburgh, Edinburgh, United Kingdom; 2 Genetics Core, Edinburgh Clinical Research Facility, Western General Hospital, University of Edinburgh, Edinburgh, United Kingdom; 3 Transgenics Core, Bioresearch & Veterinary Services, University of Edinburgh, Edinburgh, United Kingdom; 4 Department of Internal Medicine, Division of Endocrinology, Leiden University Medical Center, Leiden, the Netherlands; 5 Pamgene International, Den Bosch, the Netherlands; 6 Division of Molecular and Systems Toxicology, Department of Pharmaceutical Sciences, University of Basel, Basel, Switzerland; 7 Mass Spectrometry Core Laboratory, Wellcome Trust Clinical Research Facility, The Queen's Medical Research Institute, University of Edinburgh, Edinburgh, United Kingdom; 8 Clinical and Translational Research Institute, Newcastle University, Newcastle upon Tyne, United Kingdom; 9 Royal (Dick) School of Veterinary Studies, University of Edinburgh, Midlothian, United Kingdom

**Keywords:** Obesity, Glucocorticoid, Metabolism, Glucose, Corticosterone, Mineralocorticoid receptor, Glucocorticoid receptor, *Cbr1*, carbonyl reductase 1, LC-MS/MS, liquid chromatography tandem mass spectrometry, 20β-DHB/F, 20β-dihydrocorticosterone/dihydrocortisol, GR, glucocorticoid receptor, MR, mineralocorticoid receptor, SNPs, single nucleotide polymorphisms, PuroR, puromycin resistance, UTR, untranslated region, Tk, thymidine kinase, NeoR, neomycin resistant: RMCE, recombination-mediated cassette exchange, NEFA, non-esterified fatty acids: GTT, glucose tolerance test, ITT, insulin tolerance test, ELISA, enzyme-linked immunoassay, qPCR, quantitative polymerase chain reaction, 11β-HSD1/2, 11β-hydroxysteroid dehydrogenase type 1/2, DEGs, differentially expressed genes

## Abstract

**Objective:**

Carbonyl reductase 1 (Cbr1), a recently discovered contributor to tissue glucocorticoid metabolism converting corticosterone to 20β-dihydrocorticosterone (20β-DHB), is upregulated in adipose tissue of obese humans and mice and may contribute to cardiometabolic complications of obesity. This study tested the hypothesis that Cbr1-mediated glucocorticoid metabolism influences glucocorticoid and mineralocorticoid receptor activation in adipose tissue and impacts glucose homeostasis in lean and obese states.

**Methods:**

The actions of 20β-DHB on corticosteroid receptors in adipose tissue were investigated first using a combination of in silico, in vitro, and transcriptomic techniques and then in vivo administration in combination with receptor antagonists. Mice lacking one *Cbr1* allele and mice overexpressing *Cbr1* in their adipose tissue underwent metabolic phenotyping before and after induction of obesity with high-fat feeding.

**Results:**

20β-DHB activated both the glucocorticoid and mineralocorticoid receptor in adipose tissue and systemic administration to wild-type mice induced glucose intolerance, an effect that was ameliorated by both glucocorticoid and mineralocorticoid receptor antagonism. *Cbr1* haploinsufficient lean male mice had lower fasting glucose and improved glucose tolerance compared with littermate controls, a difference that was abolished by administration of 20β-DHB and absent in female mice with higher baseline adipose 20β-DHB concentrations than male mice. Conversely, overexpression of *Cbr1* in adipose tissue resulted in worsened glucose tolerance and higher fasting glucose in lean male and female mice. However, neither *Cbr1* haploinsfficiency nor adipose overexpression affected glucose dyshomeostasis induced by high-fat feeding.

**Conclusions:**

Carbonyl reductase 1 is a novel regulator of glucocorticoid and mineralocorticoid receptor activation in adipose tissue that influences glucose homeostasis in lean mice.

## Highlights

• Carbonyl reductase 1 (*Cbr1*) is a novel regulator of adipose glucocorticoid metabolism and glucose homeostasis.
• Cbr1 metabolises corticosterone to 20β-dihydrocorticosterone, which is both a glucocorticoid and mineralocorticoid receptor agonist.
• Global knockdown of *Cbr1* improves glucose tolerance in lean male mice.
• Overexpression of *Cbr1* in adipose tissue worsens glucose tolerance in lean male and female mice.

## 1 Introduction

Glucocorticoids act through widely expressed glucocorticoid receptors (GR) and the more tissue-specific mineralocorticoid receptor (MR) to modulate fuel metabolism, the immune system, and salt and water balance. Adipose tissue expresses both GR and MR, and the balance of activation between the two is an important determinant of adipose tissue expansion, insulin sensitivity, and glucose homeostasis [1,2]. Excessive or chronic activation of GR and/or MR in adipose tissue results in glucose intolerance and lipid accumulation and contributes to metabolic syndrome [[3], [4], [5], [6], [7]]. Glucocorticoids are the main ligands of GR, while MR binds both glucocorticoids and aldosterone. In classic MR-responsive tissues such as the kidney, aldosterone binding is favoured due to the presence of 11β-hydroxysteroid dehydrogenase type 2 (11β-HSD2), which inactivates cortisol/corticosterone. In adipose tissue, however, there is little 11β-HSD2, and glucocorticoids are the primary ligands of MR [8]. Receptor activation by glucocorticoids in adipose tissue is modulated by steroid-metabolising enzymes such as 11β-hydroxysteroid dehydrogenase type 1 (11β-HSD1) and 5α-reductases, which catalyse the conversion of primary glucocorticoids into more or less potent ligands of the receptors [9,10]. Dysregulation of these glucocorticoid-metabolising enzymes in adipose tissue directly contributes to insulin dysregulation [11,12] and can contribute to the pathogenesis of obesity and cardiovascular disease [9,13,14].

We recently showed that the cytosolic enzyme carbonyl reductase 1 (*Cbr1*) is a novel regulator of tissue glucocorticoid metabolism that converts cortisol/corticosterone into 20β-dihydrocortisol (20β-DHF) or 20β-corticosterone (20β-DHB), which are weak agonists of human and murine GR [15]. *Cbr1* and 20β-DHF/B are abundant in adipose tissue and increased in obese adipose of humans and mice [15,16]. There is growing evidence that *Cbr1* can affect metabolism and in particular glucose homeostasis. Single nucleotide polymorphisms (SNPs) in the human *CBR1* gene that increase *CBR1* expression are causally associated with higher fasting blood glucose [15] and deficiency of the key transcriptional regulator of *Cbr1,* *Nrf2*, improves glucose tolerance in murine models [17]. *Cbr1* was identified by RNA sequencing as a key gene involved in the pathogenesis of a streptozotocin-induced rat model of diabetes [18] and is significantly upregulated in the rat heart in diabetes [19]. Reduced CBR1 may contribute to the metabolic benefits of a Mediterranean diet since polyphenol constituents are inhibitors of CBR1 [20,21]. Despite this evidence, there have been no definitive intervention studies to test CBR1's role in metabolic health.

Having previously shown that 20β-DHF/B activates GR, we first tested the hypothesis that 20β-DHB is a ligand of MR in adipose tissue and that its administration impairs systemic glucose tolerance through increased GR and MR activation. We then addressed the hypothesis that global deficiency of *Cbr1* reduces plasma and tissue 20β-DHB, resulting in a reduction in GR and MR activation and improved glucose tolerance with or without high-fat feeding. We also tested the hypothesis that metabolic effects of *Cbr1* are mediated by adipose tissue using a model of adipose-specific overexpression.

## 2 Methods

### 2.1 Animals

Animal experiments were approved by the University of Edinburgh ethical committee and performed under the Provisions of the Animal Scientific Procedures Act (1986) of the UK Home Office in accordance with EU Directive 2010/63/EU.

Mice heterozygous for *Cbr1* deletion were generated; homozygosity of this gene deletion is foetal lethal [22]. A targeting vector was designed to introduce loxP sequences flanking *Cbr1* exons 2 and 3 (including the 3′ untranslated region) (Taconic Biosciences, Leverkusen, Germany). The positive selection marker (puromycin resistance, PuroR) was flanked by F3 sites and inserted downstream of the 3’ UTR. The targeting vector was generated using BAC clones from the C57BL/6J RPCI-23 BAC library and transfected into the Taconic Biosciences C57BL/6NTac embryonic stem cell line. Homologous recombinant clones were isolated using positive (PuroR) and negative (thymidine kinase, Tk) selections. The constitutive knockout allele was obtained by treating 1-cell embryos with soluble HTN-Cre enzyme (Excellgen, Rockville, MD, USA) as previously described [23,24]. The mice were genotyped by Transnetyx (Memphis, TN, USA) using real-time qPCR (RT-qPCR).

To generate adipose-specific over-expressors of *Cbr1*, animals with floxed *Cbr1* (*R26-Cbr1*^*Fl*^) were first generated (Taconic Biosciences GmBH). The following elements were inserted into the *Rosa26* locus using recombination-mediated cassette exchange (RMCE): a CAG promoter cassette, a loxP-flanked transcription termination cassette (STOP) containing a combination of polyadenylation signals, the *Cbr1-*T2A-mKate2 open reading frame together with a Kozak sequence (GCCACC), the human growth hormone (hGH) polyadenylation signal, and an additional polyadenylation signal. The RMCE vector was transfected into the Taconic Biosciences C57BL/6ES cell line equipped with RMCE docking sites in the ROSA26 locus. Recombinant clones were isolated using positive (Neomycin resistance- NeoR) selection. The adipose-specific overexpressors (*R26-Cbr1*^*Adpq*^) were obtained by crossing with *Adiponectin-Cre* mice [25]. The mice were genotyped by Transnetyx using RT-qPCR.

Male and female mice were maintained in individual ventilated cages at 21 °C on a 12-h light/12-h dark cycle with free access to food and water unless otherwise stated. The mice were given a high-fat diet (D12331, Research Diets Inc., NJ, USA) for 8 weeks. Bodyweight and food intake were measured weekly using a precision scale. Body composition was determined using time-domain nuclear magnetic resonance (Bruker, Billerica, MA, USA) before and after high-fat feeding. Mice undergoing adrenalectomy were maintained on 0.9% saline. Blood for glucocorticoid analysis was collected following decapitation between 9 am and 10 am, and the animals were not fasted.

### 2.2 Extraction and quantification of mRNA by RT-qPCR

Total RNA was extracted from adipose and liver using an RNeasy Mini kit (Qiagen Inc., Valencia, CA, USA) according to the manufacturer's instructions. The tissue was mechanically disrupted in either QIAzol (Qiagen) for adipose tissue or RLT buffer (Qiagen) for liver tissue. cDNA was synthesised using a QuantiTect Reverse Transcription kit (Qiagen) according to the manufacturer's instructions. A quantitative real-time polymerase chain reaction was performed using a LightCycler 480 (Roche Applied Science, Indianapolis, IN, USA). Primers were designed using sequences from the National Centre of Biotechnological Information and the Roche Universal Probe Library. The qPCR primer sequences are included in Supplementary Table 1. Samples were analysed in triplicate and amplification curves plotted (y axis, fluorescence; x axis, cycle number). Triplicates were deemed acceptable if the standard deviation of the crossing point was <0.5 cycles. A standard curve (y axis, crossing point; x axis, log concentration) for each gene was generated by serial dilution of cDNA pooled from different samples, fitted with a straight line, and deemed acceptable if the reaction efficiency was between 1.7 and 2.1. The average of housekeeping genes 18s, *Tbp*, and *β-actin* was used to normalise gene expression.

### 2.3 Quantification of protein by western blotting

Protein lysates from subcutaneous adipose tissue and liver (30–50 mg) were prepared in RIPA lysis buffer supplemented with protease inhibitors (Thermo Fisher Scientific, Waltham, MA, USA). The protein concentration was quantified using a bicinchoninic acid (BCA) assay (Thermo Fisher Scientific). Extracted proteins (20 μg) were resolved by SDS-PAGE using Criterion TGX Precast Protein Gels 4–20% (Bio-Rad) under reducing and denaturing conditions. Proteins were transferred to nitrocellulose membranes using the Trans-Blot Turbo Blotting System (Bio-Rad). Membranes were blocked with skim milk at 5% in Tris-buffered saline and then subjected to Western blotting using antibodies specific for *CBR1* (rabbit polyclonal IgG, cat. no. NBP1-86595, Novus Biologicals) and *β-actin* (mouse monoclonal IgM, 7D2C10, cat. no. 60008-1-Ig, Proteintech). The primary antibodies were used at 1:1000 and 1:5000 dilution in 3% BSA in Tris-buffered saline and incubated overnight (4 °C). Secondary antibodies IRDye 800CW or IRDye 680CW (LI-COR) (anti-mouse and rabbit IgGs) were used at 1:10,000 dilution in 3% BSA in Tris-buffered saline and incubated for 1 h at room temperature. Protein detection was performed using an Odyssey CLx Imaging system (LI-COR). Densitometric analyses were performed using Image Studio Software (LI-COR).

### 2.4 CBR1 activity

CBR1 activity was determined in adipose and liver homogenised in Krebs buffer as previously described [26]. Briefly, homogenates (1 mg/mL of protein) were incubated with menadione (100 nM) and NADPH (2 mM) and the absorbance measured at 340 nm at 25 °C for 45 min using a Spectra Max Plus microplate reader (Molecular Devices LLC). Activity was defined as (ΔAbs_340_/min)/6.3 and expressed per mg of protein.

### 2.5 Quantification of steroids in plasma and adipose by LC-MS/MS

Mouse plasma (100 μL) samples were prepared alongside calibration standards (covering a range of 0.025–500 ng/mL) in a 96-well plate enriched with internal standard (10 ng, d8-corticosterone, and d8-aldosterone) and diluted with 0.1% formic acid in water (100 μL) on a Biotage Extrahera liquid-handling robot. Diluted samples were transferred to an SLE+ 200 plate and eluted into a collection plate with dichloromethane/propan-2-ol (98:2; 4 × 450 μL). The eluate was dried and reconstituted in water/methanol (70:30; 100 μL) before injecting directly from the 96-well plate for LC-MS/MS analysis.

Adipose tissue samples (60–80 mg) were enriched with internal standard (0.5 ng; d8-corticosterone) homogenised (TissueLyser II, Qiagen) in acetonitrile w/0.1% formic acid (500 μL). A calibration standard curve of 20β-DHB was prepared alongside the samples covering a range of 0.0025–10 ng. The samples were centrifuged and the supernatant (500 μL) was transferred to an ISOLUTE PLD+ 96-well plate cartridge (Biotage, Uppsala, Sweden), subjected to positive pressure, collected, and dried under nitrogen gas (40 °C). The samples were re-suspended in H_2_O: MeOH (70:30; 100 μL), sealed before analysis.

Extracts were analysed by LC-MS/MS on a Shimadzu Nexera X2 connected to a QTrap 6500+ mass spectrometer (AB Sciex) adapted from earlier methods [27]. Standards and samples were injected (20 μL) onto a Kinetex C18 column (100 × 3.0 mm and 2.6 μm; Phenomenex, UK) fitted with a 0.5 μm Ultra KrudKatcher (Phenomenex) using a mobile phase system of A 0.05 mM ammonium fluoride in water and methanol at 0.5 mL/min from 50 to 90% corticosterone over 16 min. Mass transitions and retention times are detailed in the supplementary materials. Peaks were integrated using Quantitate software and the peak area ratio of 20β-DHB to d8-corticosterone using least-squares regression with 1/x weighting was used to calculate the amount of 20β-DHB in the samples, then normalised to the tissue weight expressed as ng/g of tissue.

### 2.6 Glucose and insulin tolerance tests

For glucose tolerance tests, the mice were fasted for 6 h (0800–1400 h) in clean cages and then given glucose (2 mg/g of bodyweight, 40% w/v in saline) via intraperitoneal injection. For insulin tolerance tests, the mice were fasted for 4 h (1000–1400 h) and then administered 0.75 IU/kg of insulin (Eli Lilly, Indianapolis, IN, USA) via intraperitoneal injection. Blood was collected from the tail vein immediately prior to and 15, 30, 60, 90, and 120 min after injection. Glucose was measured immediately using a point-of-care glucometer (Accu-Chek Aviva, Roche, Basel, Switzerland). Plasma insulin was measured using an Ultra-Sensitive Mouse Insulin ELISA kit (Crystal Chem Inc., Elk Grove Village, IL, USA). Plasma non-esterified fatty acids were measured by ELISA (NEFA-HR, Wako Chemicals GmBH, Neuss, Germany) following the manufacturer's instructions.

### 2.7 20β-DHF interaction with human mineralocorticoid receptor

Docking studies were performed using GOLD software version 5.2 (Cambridge Crystallographic Data Centre, Cambridge, UK) [28]. This software allows the identification of precise docking poses for small molecules in a protein's binding pocket by applying a genetic algorithm. The crystal structures with the Protein Data Bank (PDB) entry 2AA2 (DOI: 10.2210/pdb2AA2/pdb) was selected for MR. First the respective co-crystallised ligand, aldosterone, was removed from the binding pocket and re-docked into the binding site to examine whether GOLD could restore the original binding position and therefore validate the docking settings. The MR binding sites were defined by the ligand surrounded by a 6 Å region lining the active site. Protein ligand interactions determined by the docking software were further assessed using LigandScout 3.12 (Inte:Ligand GmbH, Vienna, Austria, kindly provided by Thierry Langer). Based on the chemical functionalities, geometric distances, and angles between adjacent structures, this software automatically evaluates the observed binding pattern between the protein and docked ligand [29]. A microarray assay for real-time co-regulator-nuclear receptor interaction (MARCoNI) was used to compare the quantitative and qualitative co-regulator recruitment induced when 20β-DHF (1 μM) binds with human MR with that of recruitment in response to aldosterone (1 μM) using a previously described method [30].

### 2.8 In vitro mineralocorticoid receptor activation

Human embryonic kidney cell line HEK293 cells were obtained from the European Collection of Cell Cultures (ECACC; distributor Sigma–Aldrich, St. Louis, MO, USA). The cells were grown and maintained in a humidified atmosphere (95% air, 5% CO_2_, and 37 °C) in Dulbecco's modified Eagle's medium (DMEM, Lonza Group Ltd., Basel, Switzerland) supplemented with glucose (4.5 g/L), heat-inactivated foetal bovine serum (HI-FBS) (10% v/v), penicillin (100 IU/mL), streptomycin (100 μg/mL), and l-glutamine (2 mM). The cells were seeded at 2 × 10^5^ per 35-mm well. The cells were rinsed twice with PBS and cultured in steroid-free medium for 24 h prior to experimentation. The cells were transiently transfected with 1 μg of pMMTV LTR-luciferase, 1 μg of pKC275 (encoding β-galactosidase as an internal control), and 0.05 μg of murine MR. The cells were treated with vehicle (ethanol), aldosterone (10^−14^-10^−5^ M, Sigma–Aldrich), or 20β-DHB (10^−12^-10^−5^ M, Steraloids, Newport, RI, USA) for 4 h and then lysed and luciferase and β-galactosidase activities were measured as previously described [31]. Galactosidase activity was assayed using a Tropix kit (Applied Biosystems, Foster City, CA, USA). The mean ratio of luciferase/β-galactosidase activities was calculated. Plasmids were a kind gift from K.E. Chapman, Centre for Cardiovascular Science, University of Edinburgh.

### 2.9 RNA sequencing analysis of adipose tissue

C57BL/6J male mice (8 weeks of age, n = 6/group) underwent adrenalectomy to remove endogenous steroids as previously described [32]. Seven days post-surgery, subcutaneous mini-osmotic pumps (Alzet, Cupertino, CA, USA) were implanted to deliver either vehicle (DMSO/propylene glycol), the GR agonist dexamethasone, the MR agonist aldosterone, or 20β-DHB (20 μg/day). After 7 days of treatment, subcutaneous adipose tissue was harvested post mortem and RNA extracted as previously described (Section 2.4). Total RNA samples were quantified using a Qubit 2.0 Fluorometer (Thermo Fisher Scientific) and a Qubit RNA HS assay kit. RNA integrity was assessed using an Agilent 2100 Bioanalyser System (Agilent Technologies Inc.) and Agilent RNA 6000 Nano kit. Libraries were prepared from 500 ng of each total RNA sample using a TruSeq Stranded mRNA Library kit (Illumina Inc.). cDNA was synthesised and libraries quantified. These and details on next-generation sequencing are given in the supplementary materials.

### 2.10 Statistical analysis

Analyses were performed using Prism 8 software (GraphPad, San Diego, CA, USA). All of the variables were assessed for normality using the Kolmogorov–Smirnov test. Comparisons between groups were performed using Student's t tests or the Mann–Whitney test as appropriate. Comparisons between groups at different time points and between > 2 groups were assessed by one- or two-way ANOVA with Bonferroni's post hoc test. Data are presented as mean ± SEM.

## 3 Results

### 3.1 20β-DHF/B was a full mineralocorticoid receptor agonist in vitro

**Figure 1:**
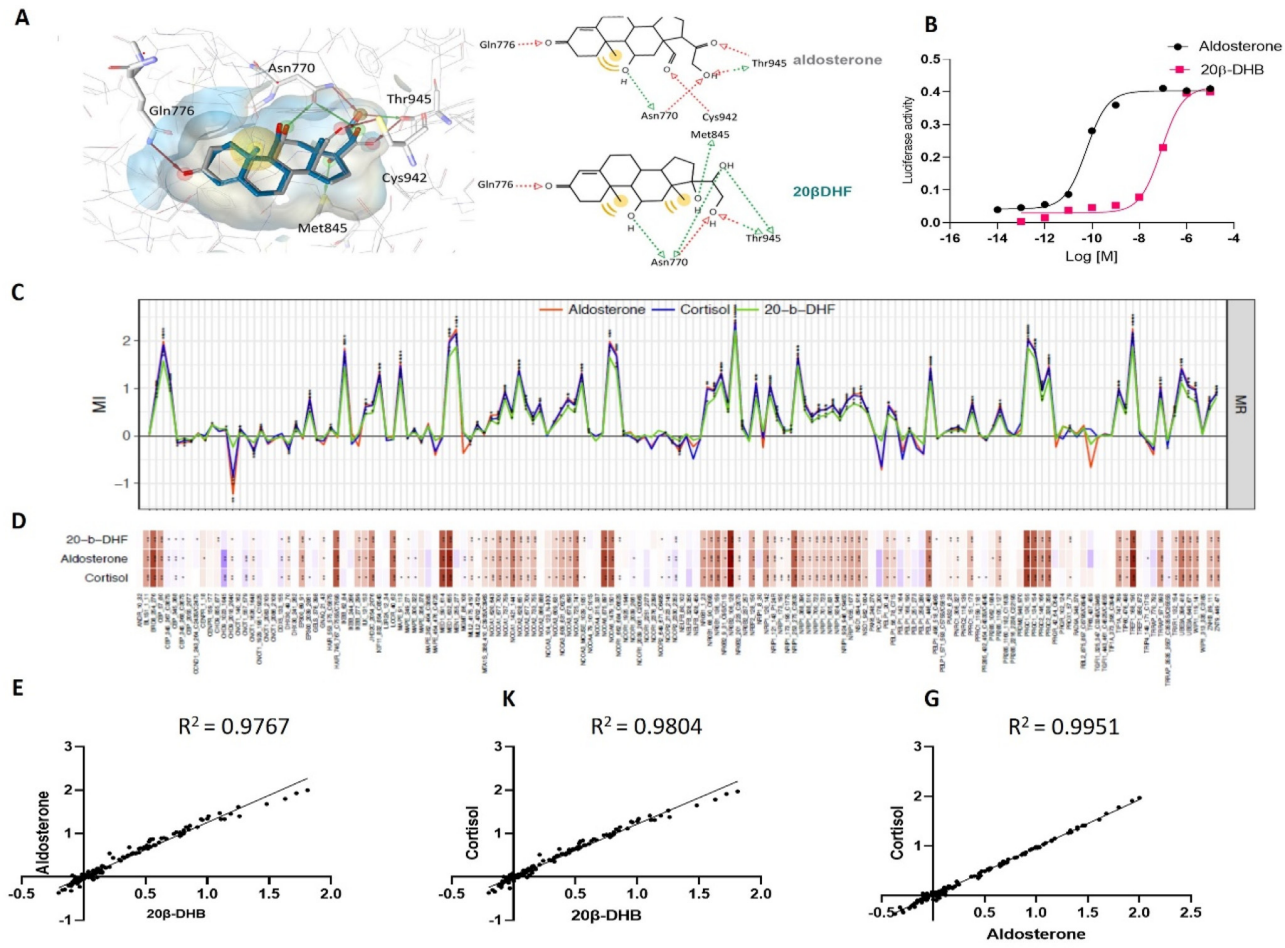
**20β-DHB is a full agonist of the mineralocorticoid receptor.** (A) In silico comparison of 20β-DHF and aldosterone binding on the human MR. (B) A dose–response curve of 20β-DHB and aldosterone in HEK293 cells transiently transfected with murine mineralocorticoid receptor. (C) Binding profile induced by aldosterone (red), cortisol (blue), or 20β-DHF (green) binding to mineralocorticoid receptor. Modulation index (MI) > 0 suggested ligand-favoured binding, while MI < 0 suggested ligand-disfavoured binding of a peptide compared to DMSO; ∗p < 0.05 and ∗∗p < 0.01. (D) Heat map depiction of details of ligand-induced binding of co-regulator peptides using MARCoNI. The colour of the bar represents the modulation index, that is, compound induced log-fold change of binding, red a positive fold change, and blue a negative fold change. (E-G) The correlation of co-regulator recruitment between 20β-DHF and aldosterone. (E) 20β-DHF and cortisol (F) and cortisol and aldosterone (G).

As there was no crystal structure of murine MR available, in silico modelling of 20β-DHF, the human equivalent to 20β-DHB, was conducted on the human MR. Docking calculations revealed similar interactions with the residues of the MR ligand-binding pocket for 20β-DHF and aldosterone. Both ligands formed hydrogen (H) bonds with Gln776, Asn770, and Thr945. The 20β-hydroxyl group on 20β-DHF formed an H bond with Met845, whereas the carbonyl group of aldosterone at the same position showed an H bond with Cys942 (Figure 1A). Based on this, we predicted that upon binding to the receptor, 20β-DHF was likely to induce a transcriptional response. This was tested in vitro using HEK293 cells transiently expressing murine MR and a luciferase reporter under the control of a promoter with a corticosteroid receptor response element. A dose–response curve showed that 20β-DHB could fully activate murine MR (20β-DHB EC50 8.5 × 10^−8^ M vs aldosterone EC50 5.7 × 10^−11^) (Figure 1B) and to a lesser extent murine GR (partial agonism at EC50 2.5 × 10^−6^ [15]). Thus, 20β-DHB was a more potent agonist of MR than GR in vitro.

On binding a ligand, the translocation to the nucleus and subsequent transcriptional response to a steroid hormone-receptor complex is largely determined by co-regulator recruitment [30]. We previously showed that on binding to GR 20β-DHF recruits only 36% of co-regulators recruited by cortisol [15]. On testing the effect of 20β-DHF on MR, we found that 20β-DHF-MR binding recruited 93% of the co-regulators recruited by the aldosterone-MR complex or cortisol-MR complex (Figure 1C–D, Supplementary File 1) and recruitment by both ligands was highly correlated (R^2^ = 0.97, p < 0.0001) (Figure 1E–G), that is to say that all of the co-regulators were recruited in the same direction by both ligands and it was only the magnitude of fold change that differed between the two. This was in line with the full agonism observed in the transactivation assay.

### 3.2 20β-DHB induced GR and MR-mediated transcriptional and functional responses in vivo

**Figure 2:**
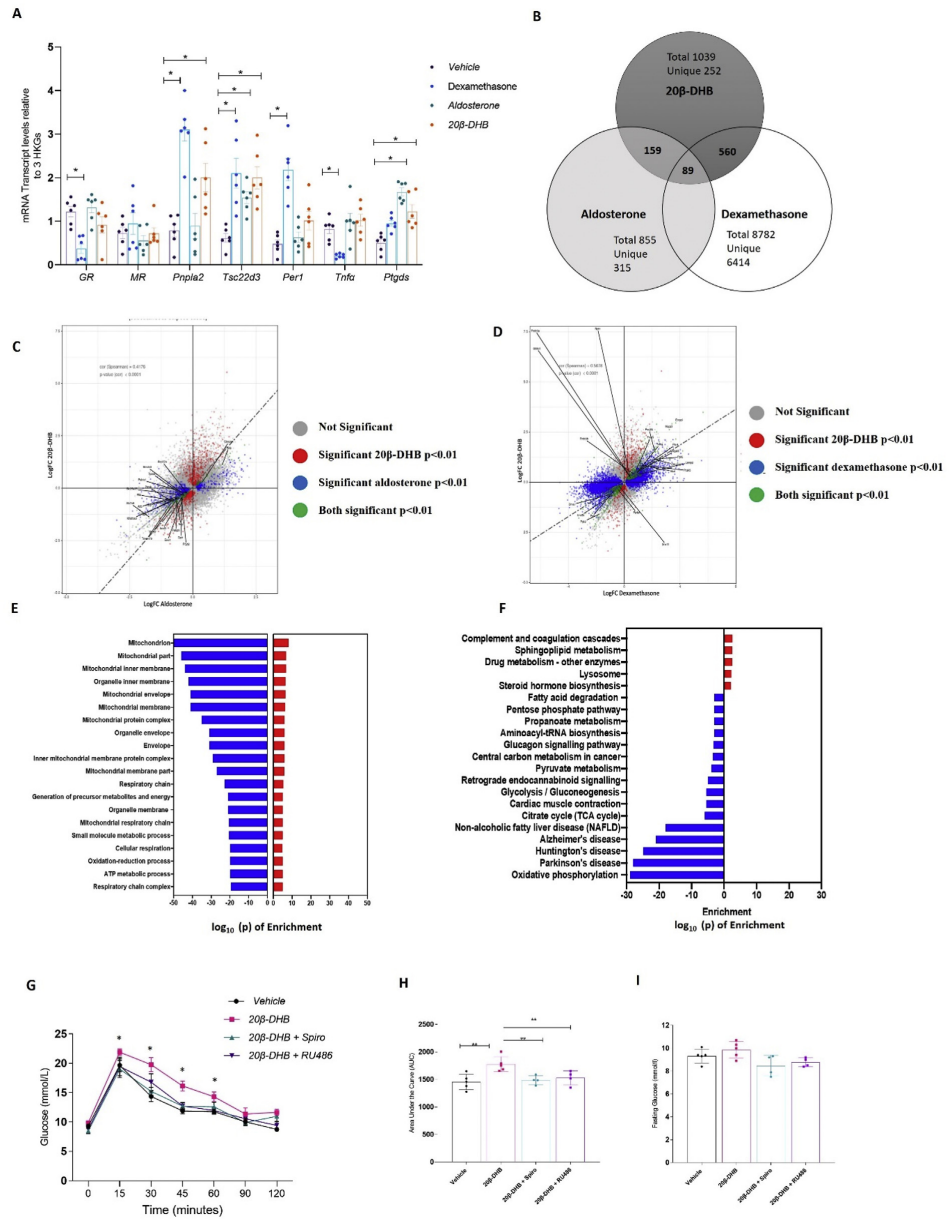
**20β-DHB induced both GR- and MR-regulated genes in adipose tissue.** (A) The mRNA expression of glucocorticoid receptor (GR), mineralocorticoid receptor (MR), GR-responsive genes patatin-like phospholipase domain-containing 2 (*Pnpla2*) that encodes adipose triglyceride lipase (Atgl), *Tsc22d3* that encodes glucocorticoid-induced leucine zipper protein (GilZ), period 1 (*Per1*), tumour necrosis factor α (*Tnfα*), and MR-responsive gene prostaglandin D2 synthase (*Ptdgs*) (n = 6/group). Data are mean ± SEM. Statistical significance was assessed by the Mann–Whitney *U* test and two-way ANOVA as appropriate. ∗p < 0.05 relative to the wild-type control. (B) Venn diagram showing overlap of significantly differentially expressed genes (DEGs) in response to aldosterone, dexamethasone, and 20β-DHB in subcutaneous adipose tissue. (C) Scatterplots of DEGs in response to 20β-DHB (red), aldosterone (blue), and both ligands (green). (D) Scatterplots of DEGs in response to 20β-DHB (red), dexamethasone (blue), and both ligands (green). (E) KEGG analysis of the 20β-DHB mediated transcriptome showing the log p value for pathway enrichment. (F) Gene ontology analysis of 20β-DHB mediated transcriptome showing the log p-value for pathway enrichment. (G) Glucose tolerance tests (GTT) in male mice administered 20β-DHB with concurrent vehicle, RU486, or spironolactone (n = 4–5 mice/group) (H) Area under the curve for GTT in the four groups. (I) Fasting plasma glucose concentrations. Data are mean ± SEM. Statistical significance was assessed by ANOVA. ∗p < 0.05 and ∗∗p < 0.01.

These in vitro findings suggested that 20β-DHB would preferentially activate MR over GR in vivo. To determine the relative activation of GR and MR by 20β-DHB, we compared the transcriptional response of subcutaneous adipose tissue to systemic infusion of either the GR agonist dexamethasone, the MR agonist aldosterone, or 20β-DHB (20 μg/day of each agonist) in wild-type mice (C57BL/6J) following adrenalectomy to remove endogenous steroids. Candidate gene expression analysis showed that dexamethasone and 20β-DHB induced transcription of GR-responsive gene *Pnpla2* and mixed GR/MR target gene *Tsc22d3* in subcutaneous adipose tissue (Figure 2A). Unlike dexamethasone, 20β-DHB did not downregulate GR or *Tnfα* (Figure 2A). The expression of MR-responsive gene *Ptgds* was increased by aldosterone and 20β-DHB (Figure 2A). Given the rarity of known MR-specific genes, we further explored the transcriptome induced by each ligand using next-generation sequencing of the subcutaneous adipose tissue.

20β-DHB differentially regulated 1039 genes (554 upregulated, 485 downregulated, Supplementary File 2), dexamethasone 8782 genes (3939 upregulated, 4843 downregulated), and aldosterone 855 (303 upregulated, 552 downregulated) (Figure 2B–D) compared with vehicle control (DMSO). 20β-DHB shared 54% of its differentially expressed genes (DEGs) with dexamethasone but this accounted for just 5% of the genes regulated by dexamethasone (561/8782), 15% with aldosterone accounting for 18% of aldosterone-regulated genes (155/855), and 8% with both; 24% were uniquely regulated by 20β-DHB. We further analysed these genes for the presence of conserved GR binding sites using oPOSSUM software [33]. Within the genes uniquely differentially regulated by 20β-DHB, 10% (26/252) had distinct GR transcription factor-binding sites (TFBS). This was similar to, but slightly less than, the percentage of TFBS identified in the genes uniquely regulated by dexamethasone (12%, 814/6414) and aldosterone (15%, 50/315). Transcriptome interrogation of the shared DEGs by KEGG and GO analysis showed that there were no significant similarities in pathway enrichment between dexamethasone and 20β-DHB or between aldosterone and 20β-DHB (data not shown). Analysis of all genes differentially regulated by 20β-DHB demonstrated significant downregulation of oxidative phosphorylation and mitochondrial pathways (Figure 2E–F and Supplementary Tables 2–3) by both GO and KEGG analysis, none of which were enriched by dexamethasone or aldosterone.

Having demonstrated that 20β-DHB activates both GR and MR in adipose tissue, male C57BL/6J mice (8 weeks of age) were administered 20β-DHB (20 μg/day) via subcutaneous mini-pumps for 7 days with concurrent administration of vehicle, GR antagonist RU486 (mifepristone, 6 mg/kg/day), or MR antagonist spironolactone (20 mg/kg/day) in drinking water. We found that administration of 20β-DHB impaired glucose tolerance in the wild-type mice and that this effect was ameliorated by antagonism of either GR or MR (Figure 2G–H). Fasting glucose was not different between the groups (Figure 2I). We selected four genes uniquely downregulated by 20β-DHB in subcutaneous adipose tissue and overrepresented in KEGG and GO analysis enrichment and determined their expression in these mice. We found that both RU486 and spironolactone normalised the expression (Supplementary Fig. S1).

### 3.3 Male but not female *Cbr1* haploinsufficient mice had reduced 20β-DHB in adipose tissue

**Figure 3:**
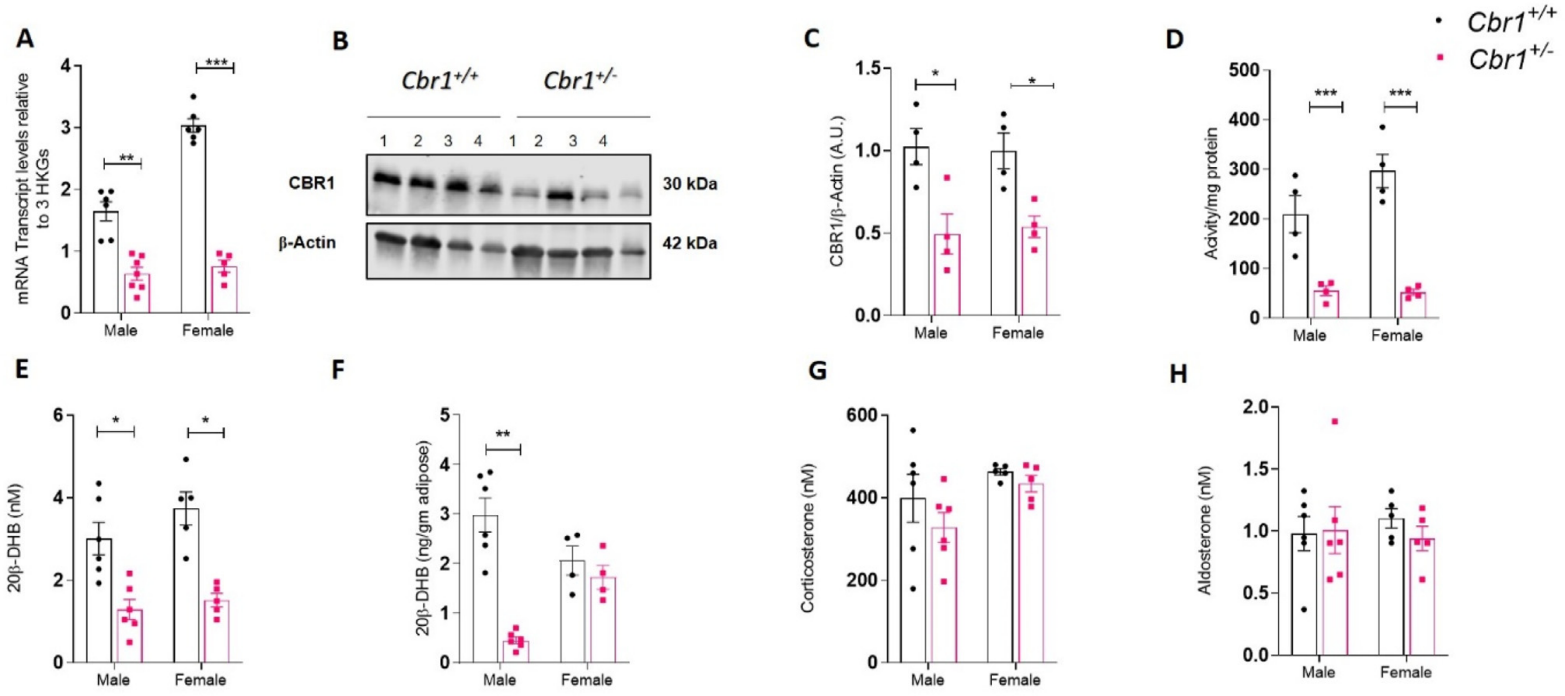
**Lean *Cbr1* heterozygous mice had reduced 20β-DHB in plasma and adipose tissue** (A) *Cbr1* mRNA expression in subcutaneous adipose tissue of lean male and female mice (n = 5–7 mice/group). (B) Representative Western blotting of CBR1 in the subcutaneous adipose tissue from the lean male *Cbr1*^+/+^ and *Cbr1*^+/−^ mice (n = 4 mice/group).**(C)**Quantification of CBR1 in the subcutaneous adipose tissue from the lean male and female *Cbr1*^+/+^ and *Cbr1*^+/−^ mice. (D) CBR1 activity in the subcutaneous adipose tissue from the lean male and female *Cbr1*^+/+^ and *Cbr1*^+/−^ mice (n = 4 mice/group). (E–F) 20β-DHB quantified by LC-MS/MS in the plasma and subcutaneous adipose tissue from the lean male and female *Cbr1*^+/+^ and *Cbr1*^+/−^ mice (n = 4–6 mice/group). (G–H) Plasma corticosterone and aldosterone quantified by LC-MS/MS in the plasma from the lean male and female *Cbr1*^+/+^ and *Cbr1*^+/−^ mice (n = 5–6 mice/group). Data are expressed as mean ± SEM. Statistical analysis was conducted with the Mann–Whitney U test. ^∗^p < 0.05, ^∗∗^p < 0.01, and ^∗∗∗^p < 0.001.

To determine the physiological effects of *Cbr1/*20β-DHB on glucose homeostasis in the lean and obese state, mice heterozygous for *Cbr1* deletion were generated (*Cbr1*^*+/−*^). Male and female control (*Cbr1*^*+/+*^) and heterozygous (*Cbr1*^*+/−*^) littermates were born at the expected Mendelian ratio but no homozygotes (*Cbr1*^*−/−*^) were born, confirming that homozygosity of this gene deletion is foetal lethal. *Cbr1* mRNA expression in the subcutaneous adipose tissue of the *Cbr1*^*+/−*^ mice was approximately 20–30% of that in their *Cbr1*^*+/+*^ control littermates (Figure 3A). CBR1 protein expression in the subcutaneous adipose of the male *Cbr1*^*+/−*^ mice was approximately 50% of that in their control littermates (Figure 3B–C). This model is globally deficient of Cbr1, which was confirmed by the gene expression and protein levels in the liver and kidney (Supplementary Fig. S2). The female mice had a similar reduction in mRNA, protein, and activity (Figure 3A–D).

Analysis of the glucocorticoid profile by LC-MS/MS demonstrated that the male *Cbr1*^*+/−*^ mice had approximately 50% less 20β-DHB in their plasma and an 80% less in their subcutaneous adipose tissue compared to their *Cbr1*^*+/+*^ control littermates (Figure 3E–F). The female mice had a similar reduction in plasma 20β-DHB concentrations but there was no difference in adipose tissue 20β-DHB content (Figure 3E–F) and adipose corticosterone content was not different between genotypes (Supplementary Fig. S3A). Plasma corticosterone and aldosterone concentrations were not different between genotypes (Figure 3G–H).

### 3.4 *Cbr1* haploinsufficiency improved glucose tolerance in lean male but not female mice but did not protect against the effects of high-fat feeding

**Figure 4:**
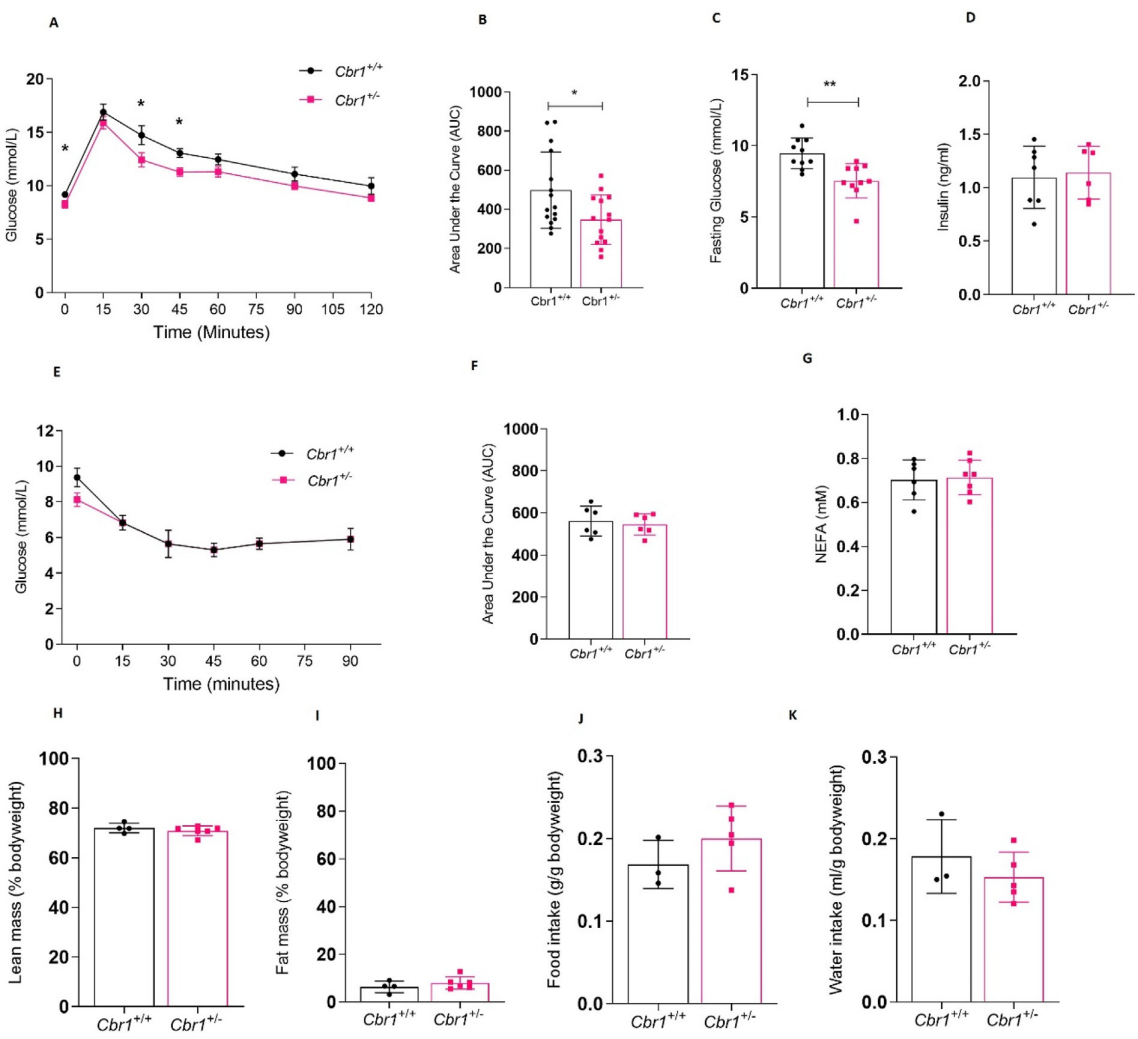
**Deletion of *Cbr1* improved glucose tolerance in the lean male mice.** (A) Glucose tolerance tests (GTT) in the *Cbr1*^+/+^ and *Cbr1*^+/−^ mice (n = 14 mice/group). (B) Area under the curve for GTT. (C–D) Fasting plasma glucose and insulin concentrations in the *Cbr1*^+/+^ and *Cbr1*^+/−^ mice (n = 6–10 mice/group). (E) Insulin tolerance test (ITT) in the *Cbr1*^+/+^ and *Cbr1*^+/−^ mice (n = 6 mice/group). (F) ITT area under the curve. (G) Fasting plasma concentrations of non-esterified fatty acid (NEFA). (H–I) Lean mass and fat mass as a percentage of bodyweight in the *Cbr1*^+/+^ and *Cbr1*^+/−^ mice (n = 4–6 mice/group). (J–K) Average food and water intake normalised to bodyweight in the *Cbr1*^+/+^ and *Cbr1*^+/−^ mice (n = 3–5 mice/group). Data are expressed as mean ± SEM. Statistical analysis was conducted with the Mann–Whitney U test. ^∗^p < 0.05, ^∗∗^p < 0.01, and ^∗∗∗^p < 0.001.

The male *Cbr1*^*+/−*^ mice had lower fasting glucose on a control chow diet and a smaller area under the curve for plasma glucose following intra-peritoneal glucose tolerance tests, indicating improved glucose tolerance compared to the *Cbr1*^*+/+*^ mice. Fasting plasma insulin, insulin tolerance, and fasting plasma non-esterified fatty acid (NEFA) did not differ between genotypes (Figure 4A–G). However, the female *Cbr1*^*+/−*^ showed no difference in fasting glucose or glucose tolerance compared to their littermate controls on a control chow diet (Supplementary Fig. S4). When fed a control chow diet (4–8 weeks of age) there were no differences between littermate controls and *Cbr1*^*+/−*^ in bodyweight, lean or fat mass, and food or water intake between the male (Figure 4H–K) or female mice (Supplementary Fig. S4).

**Figure 5:**
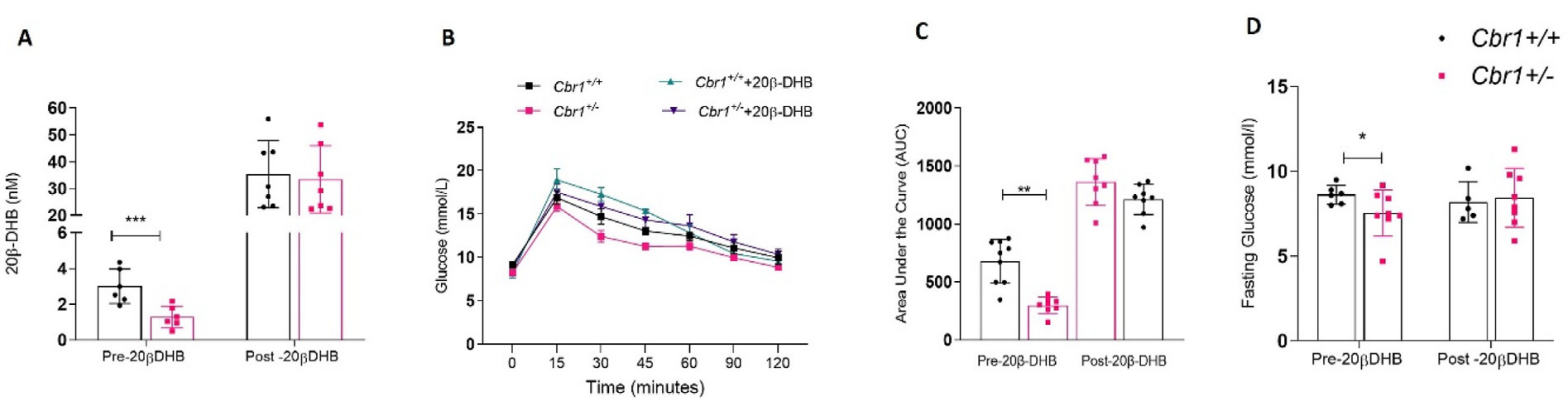
**Supplementation with 20β-DHB abolished genotype differences in glucose tolerance.** (A) Plasma 20β-DHB concentrations before and after supplementation in drinking water for 7 days in the male *Cbr1*^*+/+*^ and *Cbr1*^*+/−*^ mice (n = 6–7 mice/group). (B) Glucose tolerance tests (GTT) prior to and following 7 days of supplementation with 20β-DHB (n = 8–9 mice/group). (C) Area under the curve for GTT. (D) Fasting plasma glucose prior to and following 7 days of supplementation with 20β-DHB (n = 5–8 mice/group). Data are expressed as mean ± SEM. Statistical analysis was conducted with the Mann–Whitney U test, a two-way ANOVA, and Bonferroni's post hoc correction. ^∗^p < 0.05, ^∗∗^p < 0.01, and ^∗∗∗^p < 0.001.

We confirmed that these effects on metabolism were due to the role of *Cbr1* in glucocorticoid metabolism by administering 20β-DHB in drinking water (100 μg/mL) for 7 days to the male mice. Administration of 20β-DHB abolished the genotype difference in fasting glucose and glucose tolerance observed on a control chow diet (Figure 5).

**Figure 6:**
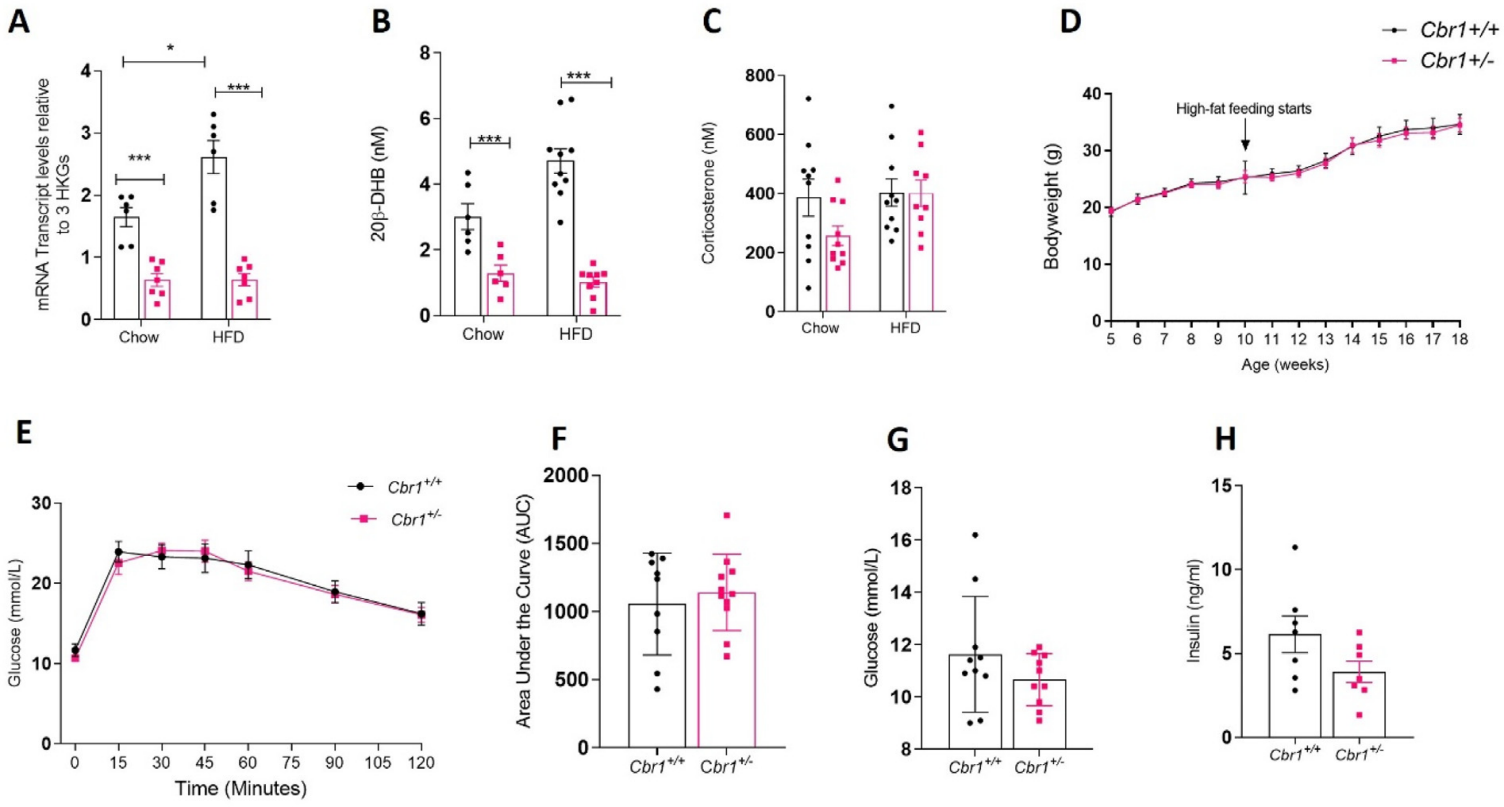
***Cbr1* deletion did not protect against the metabolic effects of high-fat feeding.** (A) mRNA expression of Cbr1 in the male *Cbr1*^*+/+*^ and *Cbr1*^*+/−*^ mice on the chow (as shown in Figure 3) and high-fat diet (HFD) (n = 6–7 mice/group). (B) Plasma 20β-DHB concentrations in the male *Cbr1*^*+/+*^ and *Cbr1*^*+/−*^ mice on chow (as shown in Figure 3) and high-fat diet (n = 6–10 mice/group). (C) Plasma corticosterone concentrations in male *Cbr1*^*+/+*^ and *Cbr1*^*+/−*^ mice on the chow (as shown in Figure 3) and high-fat diet (n = 9–10 mice/group). (D) Weight gain in the male *Cbr1*^*+/+*^ and *Cbr1*^*+/−*^ mice on the chow diet up to 10 weeks of age and then on the high-fat diet (n = 7–10 mice/group). (E) Glucose tolerance tests (GTT) in the male *Cbr1*^*+/+*^ and *Cbr1*^*+/−*^ mice following 8 weeks of high-fat feeding (n = 9–11 mice/group). (F) Area under the curve for GTT. (G) Fasting glucose concentrations in the male *Cbr1*^*+/+*^ and *Cbr1*^*+/−*^ mice following 8 weeks of high-fat feeding (n = 10 mice/group). (H) Fasting insulin concentrations in the male *Cbr1*^*+/+*^ and *Cbr1*^*+/−*^ mice following 8 weeks of high-fat feeding (n = 7 mice/group). Data are expressed as mean ± SEM. Statistical analysis was conducted with two-way ANOVA and Bonferroni's post hoc correction. ^∗^p < 0.05, ^∗∗^p < 0.01, and ^∗∗∗^p < 0.001.

We previously showed that *Cbr1* increased in adipose tissue in mice on a high-fat diet. To determine the effect of *Cbr1* deletion in obesity, the male mice were fed a high-fat diet for 8 weeks. Transcript levels and plasma 20β-DHB concentrations were still reduced in the *Cbr1*^+/−^ mice compared with their littermate *Cbr1*^*+/+*^ controls but plasma corticosterone was not different (Figure 6A–C). Weight gain, lean mass, and fat mass did not differ between the genotypes following high-fat feeding in the males (Figure 6D) or females (Supplementary Fig. S4). High-fat feeding of the male mice abolished the difference between genotypes in fasting glucose and glucose tolerance; fasting insulin remained similar (Figure 6E–H). The female *Cbr1*^*+/−*^ mice continued to demonstrate no significant differences in metabolic parameters on a high-fat diet compared with their littermate controls (Supplementary Fig. S4).

### 3.5 *Cbr1* overexpression in adipose tissue increased adipose 20β-DHB concentrations

**Figure 7:**
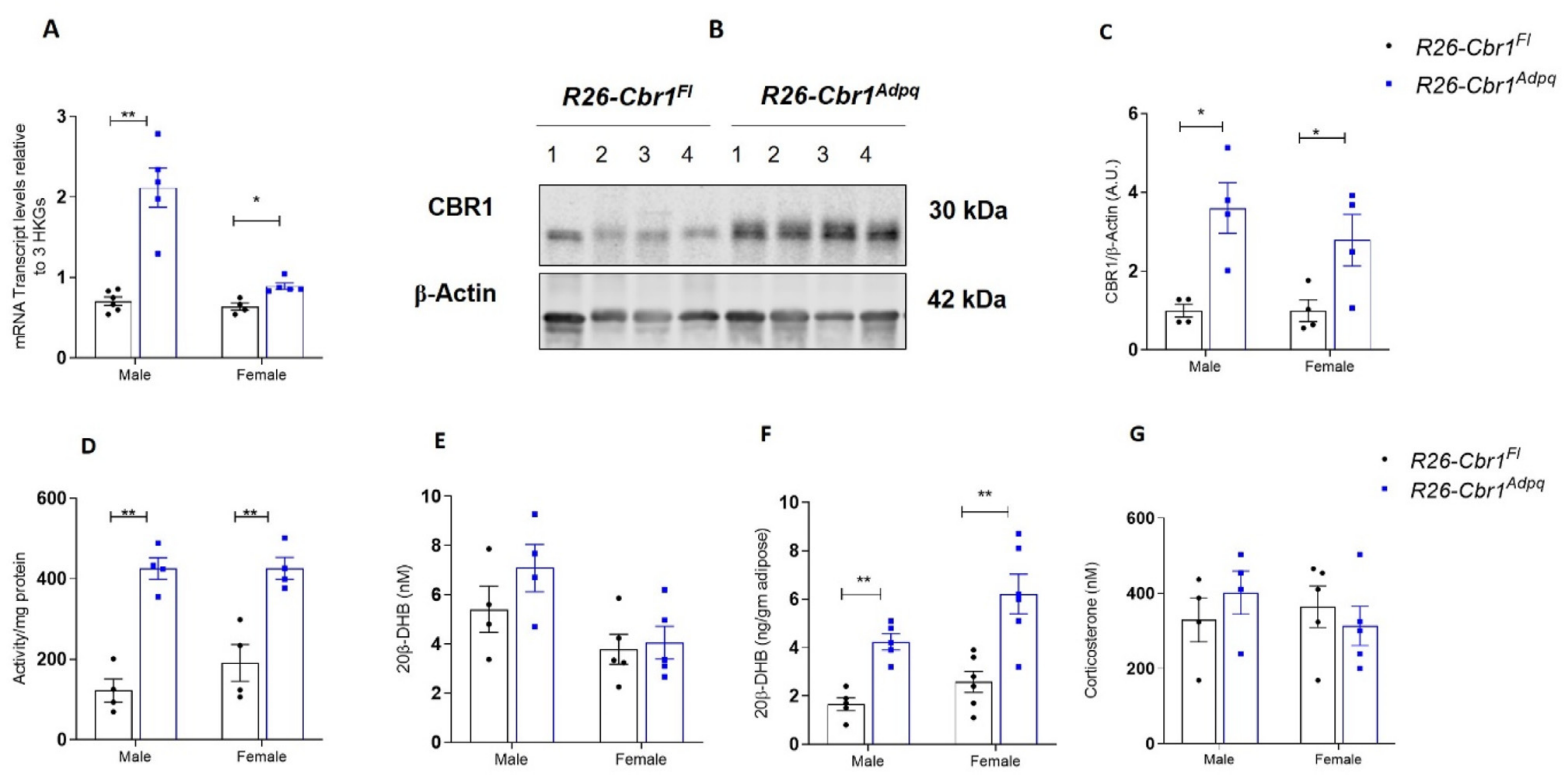
**Mice overexpressing *Cbr1* in adipose tissue had increased 20β-DHB in adipose tissue but not plasma.** (A) *Cbr1* mRNA expression in the subcutaneous adipose tissue of the male and female mice (n = 4–6 mice/group). (B) Representative Western blotting of CBR1 in the subcutaneous adipose tissue from the male *R26-Cbr1*^*Fl*^ and *R26-Cbr1*^*Adpq*^ mice (n = 4 mice/group). (C) Quantification of CBR1 in the subcutaneous adipose tissue from the male and female *R26-Cbr1*^*Fl*^ and *R26-Cbr1*^*Adpq*^ mice. (D) CBR1 activity in the subcutaneous adipose tissue from the male and female *R26-Cbr1*^*Fl*^ and *R26-Cbr1*^*Adpq*^ mice (n = 4 mice/group). (E–F) 20β-DHB quantified by LC-MS/MS in the plasma and subcutaneous adipose tissue from the male and female *R26-Cbr1*^*Fl*^ and *R26-Cbr1*^*Adpq*^ mice (n = 4–6 mice/group). (G) Plasma corticosterone quantified by LC-MS/MS in the plasma from the male and female *R26-Cbr1*^*Fl*^ and *R26-Cbr1*^*Adpq*^ mice (n = 4–5 mice/group). Data are expressed as mean ± SEM. Statistical analysis was conducted with the Mann–Whitney U test and two-way ANOVA. ^∗^p < 0.05 and ^∗∗^p < 0.01.

To test the hypothesis that adipose tissue *Cbr1* specifically mediates the effects on systemic glucose tolerance, we generated adipose-specific over-expressors of *Cbr1* (*R26-Cbr1*^*Adpq*^) by crossing conditional knock-in mice with adiponectin-Cre mice. The mKate expression, used as a surrogate for recombination, was only detected in the adipose tissue of the *R26-Cbr1*^*Adpq*^ mice and not in *R26-Cbr1*^*Fl*^ (data not shown). The mRNA expression of *Cbr1* was approximately 60% higher in the *R26-Cbr1*^*Adpq*^ male mice than in their floxed littermate controls but only 20% higher in the female *R26-Cbr1*^*Adpq*^ mice (Figure 7A). Protein and activity were approximately doubled in the male and female *R26-Cbr1*^*Adpq*^ mice compared with floxed controls (Figure 7B–C). The *R26-Cbr1*^*Adpq*^ mice had approximately twice as much 20β-DHB in their subcutaneous adipose tissue as the floxed littermate controls (1.6 vs 4.2 ng/g adipose, p = 0.0003) (Figure 7D–F). Adipose corticosterone content was not different between genotypes (Supplementary Fig. S3B). There were no differences in the plasma 20β-DHB or corticosterone concentrations (Figure 7G). There was no increase in mRNA expression in the liver of the *R26-Cbr1*^*Adpq*^ mice compared with controls (Supplementary Fig. S5).

### 3.6 Adipose-specific overexpression of *Cbr1* worsened metabolic status in lean mice but did not exacerbate the effects of high-fat feeding

**Figure 8:**
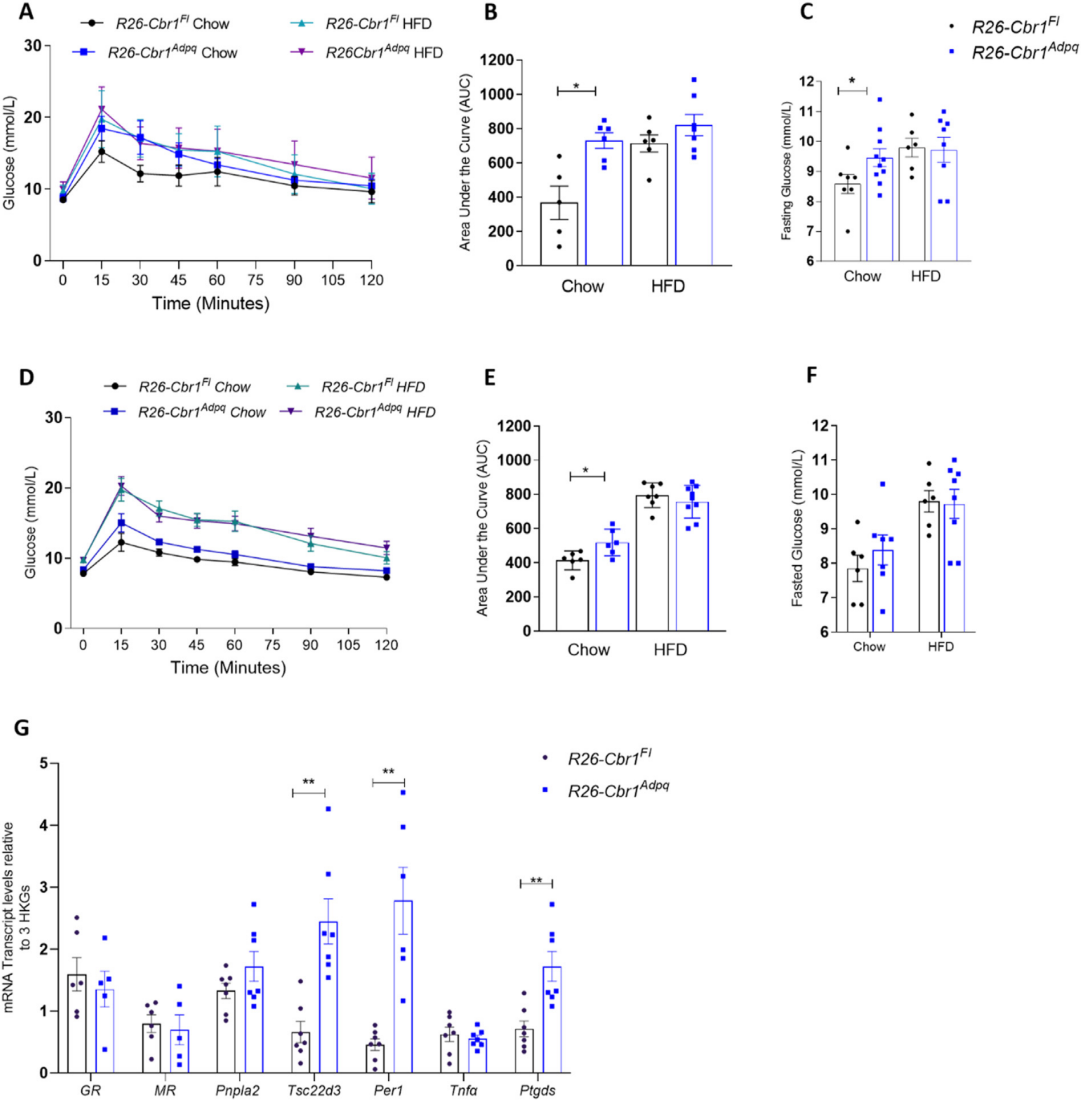
**Adipose-specific overexpression of *Cbr1* worsened metabolic status on a chow diet but did not exacerbate the effects of high-fat feeding.** (A) Glucose tolerance tests (GTT) in the male *R26-Cbr1*^*Fl*^ and *R26-Cbr1*^*Adpq*^ mice on a chow diet and after high-fat feeding (n = 5–7 mice/group). (B) Area under the curve for GTT in the male mice. (C) Fasting plasma glucose concentrations in the male *R26-Cbr1*^*Fl*^ and *R26-Cbr1*^*Adpq*^ mice (n = 6–10 mice/group). (D) Glucose tolerance tests (GTTs) in the female *R26-Cbr1*^*Fl*^ and *R26-Cbr1*^*Adpq*^ mice on a chow diet and after high-fat feeding (n = 6–9 mice/group). (E) Area under the curve for GTTs in the female mice. (F) Fasting plasma glucose concentrations in the female *R26-Cbr1*^*Fl*^ and *R26-Cbr1*^*Adpq*^ mice (n = 6–8 mice/group). (G) The mRNA expression of glucocorticoid receptor (GR), mineralocorticoid receptor (MR), GR-responsive gene patatin-like phospholipase domain containing 2 (*Pnpla2*) that encodes adipose triglyceride lipase (Atgl), *Tsc22d3* that encodes glucocorticoid-induced leucine zipper protein (*Gilz*), period 1 (*Per1*), tumour necrosis factor α (*Tnfα*), and MR-responsive gene prostaglandin D2 synthase (*Ptdgs*) in the subcutaneous adipose tissue of the male *R26-Cbr1*^*Fl*^ and *R26-Cbr1*^*Adpq*^ mice on a chow diet (n = 6–7/group). Data are mean ± SEM. Statistical significance was assessed by the Mann–Whitney U test and ANOVA. ∗p < 0.05 and ∗∗p < 0.01.

When fed a control chow diet, there were no differences in bodyweight or lean or fat mass between the male or female *R26-Cbr1*^*Adpq*^ and their floxed littermate controls (Supplementary Fig. S6). The male *R26-Cbr1*^*Adpq*^ mice had higher fasting glucose on a chow diet (9.5 ± 0.3 vs 8.4 ± 0.3, p = 0.04) and a larger area under the curve of plasma glucose following intra-peritoneal glucose tolerance tests (1819 ± 66 vs 1392 ± 14, p = 0.03) (Figure 8A–C). The female *R26-Cbr1*^*Adpq*^ mice had a larger area under the curve of plasma glucose following intra-peritoneal glucose tolerance tests but fasting glucose was not different between the genotypes (Figure 8D–F). Fasting insulin and fasting NEFA did not differ between the genotypes in either sex on either diet (Supplementary Fig. S6). High-fat feeding of the male and female mice abolished the difference between genotypes in fasting glucose and glucose tolerance (Figure 8A–F). The mRNA expression of GR- and MR-responsive genes in the subcutaneous adipose tissue showed a similar pattern to that seen when 20β-DHB was administered to adrenalectomised mice (Figure 8G and Figure 2A). The GR and MR levels were unaltered. GR-induced genes *Pnpla2*, *Tsc22d3*, and *Per1* increased in *R26-Cbr1*^*Adpq*^. *Tnfα*, which is downregulated by GR activation, was unaltered by the genotype (Figure 8G). The expression of MR-responsive gene *Ptgds* also increased in *R26-Cbr1*^*Adpq*^ compared with floxed controls (Figure 8G). These results were consistent with mixed GR and MR activation in adipose tissue.

## 4 Discussion

In this study, we showed that carbonyl reductase 1 is a novel regulator of glucocorticoid and mineralocorticoid receptor activation in adipose tissue with a role in regulating glucose homeostasis. We showed that 20β-DHB activates both GR and MR in adipose tissue but has a transcriptional profile that is distinct from either dexamethasone or aldosterone and characterised by the downregulation of oxidative phosphorylation and mitochondrial pathways. Furthermore, we demonstrated that systemic administration of 20β-DHB worsens glucose tolerance and this effect is ameliorated by antagonism of both GR and MR. We found that *Cbr1* haploinsufficiency improves glucose tolerance and lowers fasting glucose, but only when accompanied by a concurrent reduction in adipose and plasma 20β-DHB concentrations as seen in the male but not female mice. Restoring 20β-DHB levels without altering corticosterone levels “rescued” the phenotype of the *Cbr1*^+/−^ mice. The importance of adipose CBR1/20β-DHB in mediating the metabolic effects of *Cbr1* was further supported by the phenotype observed in mice with adipose-specific overexpression of *Cbr1*, with both males and females demonstrating increased levels of 20β-DHB in adipose but not plasma and both sexes having worsened glucose tolerance. Unlike other models of altered glucocorticoid action in adipose tissue, the influence of adipose CBR1/20β-DHB on glucose tolerance was not associated with altered fat mass or bodyweight and was attenuated by high-fat feeding, suggesting that 20β-DHB modulates a different balance of effects compared with “conventional” GR ligands.

Sex-specific effects of *Cbr1* manipulation were previously reported in studies investigating the role of *Cbr1* in doxorubicin metabolism. Freeland et al. observed that female *Cbr1*^*+/−*^ mice were not protected against the cardiotoxic effects of doxorubicin to the same extent as male *Cbr1*^*+/−*^ mice and suggested that this was because tissue *Cbr1* levels were higher in wild-type females than males, although they did not report enzyme activity [34]. Differences in *CBR1* expression in humans are thought to account for the increased susceptibility of women [35] and ethnic minorities to doxorubicin toxicity [36]. In our study, *Cbr1* mRNA expression and activity were higher in the adipose tissue in the control females than males but haploinsufficiency resulted in similarly low levels of mRNA expression and activity in the males and females and was therefore unlikely to account for the differences between the sexes. Explanations for the disparity between adipose *Cbr1* and 20β-DHB levels in the females include the following possibilities: CBR1 is not the only enzyme responsible for 20β-DHB production in females, removal of 20β-DHB from the adipose differs between sexes, or in female adipose, CBR1 always favours corticosterone as a substrate whereas in males, competitive or alternative substrates are available that are preferentially metabolised when CBR1 levels are reduced. The female *Cbr1*^*+/−*^ mice had a reduction in plasma concentrations of 20β-DHB but did not have a reduction in adipose tissue concentrations, suggesting that other tissues, such as the gut, may contribute more 20β-DHB to the circulating pool than adipose, supported by the finding that plasma levels were not increased in the adipose-specific *Cbr1* overexpressing mice. The fact that adipose 20β-DHB and glucose tolerance was unaltered in the female *Cbr1*^*+/−*^ supported the hypothesis that adipose 20β-DHB was the driver of the phenotype observed in the males. This was further supported by the presence of increased 20β-DHB levels in the adipose tissue of the female *R26-Cbr1*^*Adpq*^ mice and worsened glucose tolerance.

It appears from this study that CBR1/20β-DHB modulates systemic glucose tolerance via a paracrine effect in adipose tissue and not by endocrine signalling from adipose to other tissues. A reduction in plasma 20β-DHB when not accompanied by a reduction in adipose 20β-DHB as in the female *Cbr1*^*+/−*^ mice was not associated with an improvement in glucose tolerance. Conversely, in the *R26-Cbr1*^*Adpq*^ mice, worsened glucose tolerance was present without a change in plasma 20β-DHB. Administering 20β-DHB systemically resulted in plasma concentrations of more than 10 times the physiological concentrations but the change in glucose tolerance had a similar magnitude to that seen with only a doubling of adipose 20β-DHB concentrations in the *R26-Cbr1*^*Adpq*^ mice. It is well documented that manipulating glucocorticoid action in adipose tissue can have systemic effects on metabolic parameters, for example, mice over-expressing 11β-HSD1 in adipose tissue have glucose intolerance but unlike *Cbr1* over-expressors, they also demonstrate increased fat mass and free fatty acids [3]. The difference in phenotype between these two models, both of which increase corticosteroid receptor activation, is most likely due to ligand-specific transcriptional responses when receptors are activated by 20β-DHB. It may also be due to a particular balance of GR and MR activation induced by 20β-DHB, in which our data suggest relatively potent MR activation; the interaction of GR and MR in determining metabolic parameters is complex and still incompletely understood with conflicting data from genetic models manipulating each receptor [1,2,7,37,38].

Glucocorticoids acting on GR and MR have diverse effects on adipose tissue biology, including reducing glucose uptake, increasing lipolysis, and regulating inflammation and adipokine release. Our study showed that 20β-DHB is a ligand of both receptors and although our in vitro data suggested that there would be a preferential activation of MR, the in vivo investigations made it apparent that the most pronounced functional effects on glucose tolerance were ameliorated by antagonism of both GR and MR. Impairment of glucose tolerance by decreasing glucose uptake and metabolism is induced by excess dexamethasone activating GR, excess corticosterone activating both GR and MR, and overexpression of MR in adipose tissue [[4], [5], [6], [7]]. It has also been shown that combined antagonism of GR and MR improves glucose tolerance [39,40]; the effect of 20β-DHB demonstrated herein is therefore consistent with that of a mixed GR/MR agonist. Interestingly, the effects of dexamethasone on glucose uptake are more marked under basal conditions compared with insulin-stimulated conditions, which may also be the case for 20β-DHB given that overexpression of *Cbr1* did not worsen glucose tolerance during high-fat feeding [41]. We found no evidence of an effect of CBR1/20β-DHB on lipolysis or inflammatory markers within the adipose tissue. *Tnfα* expression was not downregulated by 20β-DHB and there were no inflammatory pathways significantly downregulated by 20β-DHB. That 20β-DHB has divergent effects compared with other glucocorticoids and aldosterone is unsurprising given that these receptors induce ligand-specific effects [42]. The distinct nature of the transcriptional response is in part due to co-regulator recruitment [43] and although 20β-DHF on binding to MR recruited almost 93% of the co-regulators recruited by binding aldosterone or cortisol it is clear that even very small differences in co-regulator recruitment can result in a marked difference in transcriptional response [44]. Moreover, the assay that we used only probes co-regulator interactions with the receptor ligand-binding domain and not the N-terminal part of the receptor. Our transcriptomic analysis demonstrated that while a significant number of 20β-DHB-associated DEGs were shared with dexamethasone (54%), only 15% were shared with aldosterone and there was no commonality in pathway enrichment between the ligands. We would therefore suggest that 20β-DHB induces a ligand-specific response when binding adipose GR and MR due to the large (GR) or subtle (MR) differences in co-regulator recruitment [45] or potentially the formation of heterodimers of receptors, which was not investigated in this study [46].

That *Cbr1* deletion did not protect from the effects of a high-fat diet is somewhat surprising, particularly given the increase in *Cbr1* observed in obese adipose in humans and mice [15]. One explanation for this is the role of CBR1 in the context of oxidative stress [47]. CBR1 is upregulated in oxidative stress; it inactivates highly reactive lipid aldehydes [47], ameliorates lipid peroxidation [48], and when overexpressed in hepatic cell lines confers protection against reactive oxygen species-induced cell damage [49]. CBR1 in pancreatic β cells appears to attenuate apoptosis and increase cell survival and insulin secretion in vitro under glucotoxic conditions [50]. Therefore, under normal diet conditions, a lack of CBR1 may be beneficial due to a reduction in GR/MR activation by 20β-DHB, but when oxidative stress increases in obesity, its absence is detrimental, cancelling out any protective effects. Equally, while overexpression in the adipose of lean mice may be detrimental due to chronic activation of GR/MR, it is likely to be beneficial when adipose expands and reactive oxygen species increase. Alternative endogenous substrates of CBR1 include prostaglandins [47,51], and the foetal lethality of *Cbr1*^*−/−*^ mice has been attributed to altered prostaglandin degradation in the amnion or uterus [22]. High-fat feeding is associated with inflammation and increased prostaglandins in adipose tissue [52]. Reduction in CBR1 may result in excess prostaglandin content within the adipose tissue, which mitigates the protective effect of reduced GR/MR activation.

In conclusion, we have for the first time described CBR1/20β-DHB as a novel mediator of glucocorticoid action in adipose tissue with a significant impact on systemic glucose homeostasis in the lean state. There is a very wide variation in *CBR1* expression in human populations [[53], [54], [55]]; common environmental factors such as cigarette smoke upregulate the enzyme [56] and CBR1 inhibitors are present in many foods and supplements [57]. Our data suggest that these variations may have important consequences for an individual's glucocorticoid metabolism and metabolic health and that these consequences should be considered when manipulating CBR1 for other reasons such as cancer treatment.

## Author contributions

Conceptualisation: R.A.M., B.R.W., M.N., A.O., and O·C.M. Methodology: L.M., A.F., A.C., M.G.F·S., M.V·K., E.A., R.H., S.G.D., and N.Z.M.H. Investigation: R.M.B·B., E.V., A.M.C., P.L., S.G.D., K.R.B., and R.H. Writing original draft: R.A.M. and R.M.B.B. Writing, review, and editing: R.A.M., R.M.B·B., B.R.W., M.N., A.O., and E.V. Funding acquisition: R.A.M. and B.R.W. Resources: R.H., A.O., L.M., and M.G.F.S. Supervision: R.A.M., B.R.W., and M.N.
