# Supplementary material for: Carbonyl reductase 1 amplifies glucocorticoid action in adipose tissue and impairs glucose tolerance in lean mice
Source: Mol Metab. 2021 Mar 27;48:101225. doi: 10.1016/j.molmet.2021.101225 (PMC8095185; doi:10.1016/j.molmet.2021.101225)
Supplement: Supplementary file 3 — Multimedia component 3 [file mmc3.docx]

**Supplementary methods**

**S1.1 Quantification of steroids in plasma by LC-MS/MS**

The mass spectrometer was operated at 600^o^C with polarity switching in multiple reaction modes in negative mode (-4.5 kV) for aldosterone at *m/z* 359.0 → 188.9 at -24 V, 359.0 → 331.1 at -22 V and d8Aldo at *m/z* 367.1 → 193.9 at -26 V and in positive mode (5.5 kV) for B, 20β-DHB at *m/z* 347.1 → 121.1 at 29 V, 347.1 → 90.0 at 75 V, 365.2 → 269.1 at 25 V, and 365.2 → 121.0 at 33V and *m/z* 355.3 → 125.1 at 31 V for d8-B. They were eluted for 2.3, 2.3, 2.5, 3.4, and 3.3 min.

**S1.2 Quantification of steroids in adipose by LC-MS/MS**

The mass spectrometer was operated in the positive ion multiple reaction mode for 20β-DHB and d8-B at 349.1 --> 91.1 at 71 V and 349.1 --> 121.1 at 33 V, and 355.3 -->125.1 at 31V, respectively. They were eluted for 2.5 and 3.3 min.

**S1.3 RNA sequencing**

Total RNA samples were quantified using a Qubit 2.0 Fluorometer (Thermo Fisher Scientific) and Qubit RNA HS assay kit. RNA integrity was assessed using an Agilent 2100 Bioanalyser System (Agilent Technologies Inc) and Agilent RNA 6000 Nano kit. Libraries were prepared from 500 ng of each total RNA sample using a TruSeq Stranded mRNA Library kit (Illumina) according to the provided protocol. Poly-A containing mRNA molecules was purified using poly-T oligo attached magnetic beads. Following purification, the mRNA was fragmented using divalent cations under elevated temperatures and primed with random hexamers. Primed RNA fragments were reverse transcribed into first-strand cDNA using reverse transcriptase and random primers. RNA templates were removed and a replacement strand was synthesised incorporating dUTP in place of dTTP to generate ds cDNA. The incorporation of dUTP in second-strand synthesis quenched the second strand during amplification as the polymerase used in the assay was not incorporated past this nucleotide. AMPure XP beads (Beckman Coulter) were then used to separate the ds cDNA from the second-strand reaction mix, providing blunt-ended cDNA. A single A nucleotide was added to the 3' ends of the blunt fragments to prevent them from ligating to another during the subsequent adapter ligation reaction, and a corresponding single T nucleotide on the 3' end of the adapter provided a complementary overhang for ligating the adapter to the fragment. Multiple indexing adapters were then ligated to the ends of the ds cDNA to prepare them for hybridisation onto a flow cell before 12 cycles of PCR were used to selectively enrich those DNA fragments that had adapter molecules on both ends and amplify the amount of DNA in the library suitable for sequencing. Amplification libraries were purified using AMPure XP beads. The libraries were quantified by fluorometry using a Qubit dsDNA HS assay and assessed for quality and fragment size using an Agilent Bioanalyser with a DNA HS kit. Sequencing was performed using a NextSeq 500/550 High-Output v2 (150 cycle) kit on the NextSeq 550 platform (Illumina). Then 48 libraries were combined in three equimolar pools of 16 based on the library quantification results and each pool was run across a single high-output flow cell.

The quality of the raw sequencing data was verified using FASTQC (http://www.bioinformatics.babraham.ac.uk/projects/fastqc/). Reads were aligned to the mouse genome (GRCm38) using STAR (<https://academic.oup.com/bioinformatics/article/29/1/15/272537>) and gene counts were quantified based on the GENCODE M18 annotation.

The data were normalised using trimmed mean of M values (TMM) (<https://genomebiology.biomedcentral.com/articles/10.1186/gb-2010-11-3-r25>) and transformed with voom (<https://genomebiology.biomedcentral.com/articles/10.1186/gb-2014-15-2-r29>), resulting in log_2_ counts per million (log-CPM) values with associated precision weights. Outlier detection of samples was performed using a combination of objective scoring methods: Hoeffding's D statistic, the mean Pearson correlation with other samples, the sum of the Euclidean distance to other samples, and the Kolmogorov-Smirnov test statistic. No quality issues were identified within the data. The statistical analysis was subsequently performed using empirical Bayes from the limma R package (<https://academic.oup.com/nar/article/43/7/e47/2414268>) using the normalised log-CPM values with associated precision weights from voom. The statistical models evaluated each treatment relative to the DMSO control. Genes were considered significantly differentially expressed at p < 0.01 without adjustment for multiple testing. Following the identification of putatively differentially expressed genes, the Kyoto Encyclopaedia of Genes and Genomes (KEGG) pathways [1] and Gene Ontology (GO) terms [2] were assessed for pathway enrichment using a hypergeometric test. Up- and downregulated genes were evaluated separately and the GO terms analysis was performed across all three GO ontologies. KEGG pathways and GO terms were considered significant at p < 0.01 from hypergeometric testing.

[1] Kanehisa, M., Goto, S., 2000. KEGG: Kyoto Encyclopedia of Genes and Genomes. Nucleic Acids Research 28(1):27-30.

[2] Ashburner, M., Ball, C.A., Blake, J.A., Butler, H., Cherry, J.M., Eppig, J.T., et al., 2001. Creating the Gene Ontology resource: Design and implementation. Genome Research 11(8):1425-1433.

**Supplementary figures**

**Figure S1**

**
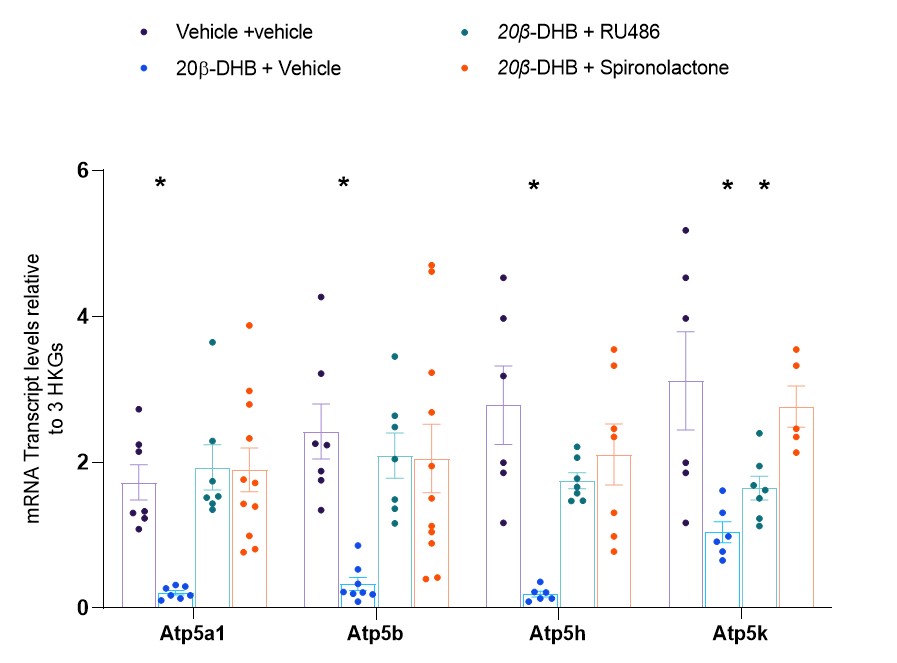
**

**Figure S1: mRNA transcript levels of selected 20β-DHB differentially regulated genes in subcutaneous adipose in mice treated with 20β-DHB with GR antagonist RU486 or MR antagonist spironolactone.** *p < 0.05 compared with vehicle. 20β-DHB downregulated genes encoding ATP synthase subunits 5a1, 5b, 5h, and 5k. Treatment with RU486 and spironolactone normalised the expression of these genes relative to the vehicle control.

**Figure S2**

**
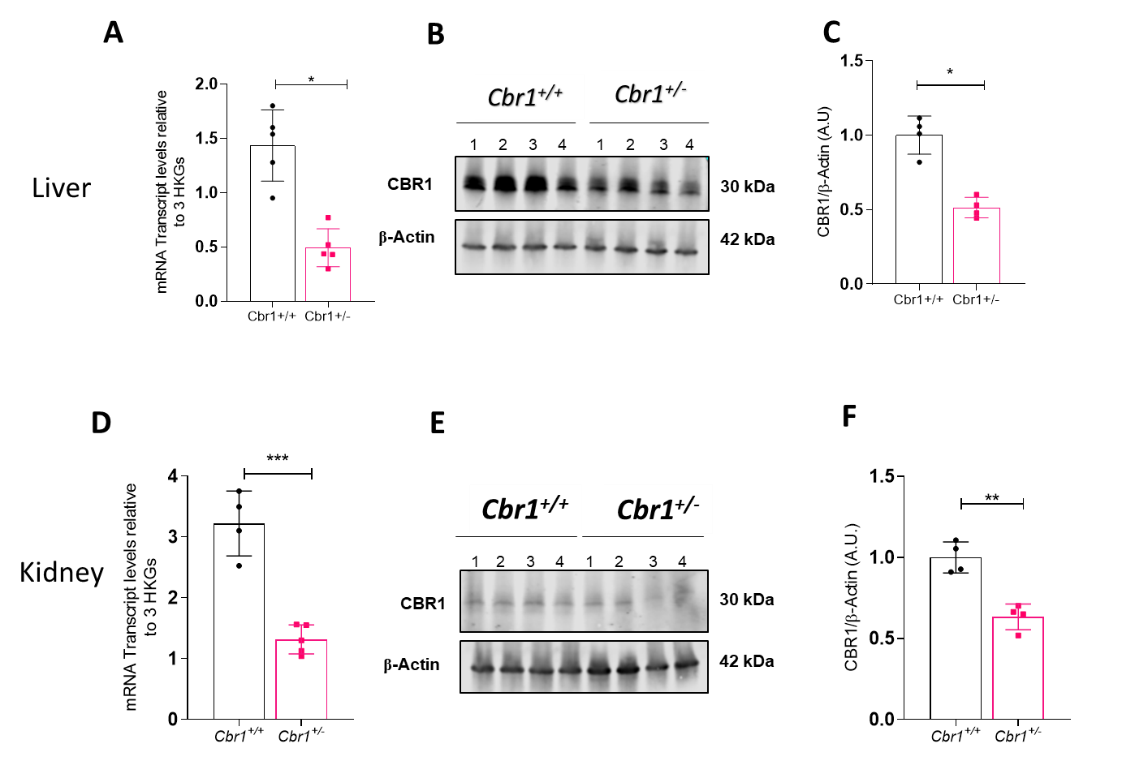
**

**Figure S2: Male *Cbr1* heterozygous mice had reduced** **CBR1 in liver and renal tissue.** (A) Cbr1 mRNA expression in hepatic tissue of the male mice (n = 5 mice/group). (B) Representative Western blotting of CBR1 in the liver from the male *Cbr1^+/+^* and *Cbr1^+/-^* mice (n = 4 mice/group). (C) Quantification of CBR1 in the liver from the male *Cbr1^+/+^* and Cbr1+/- mice (D) Cbr1 mRNA expression in the renal tissue of the male mice (n = 4 mice/group). (E) Representative Western blotting of CBR1 in the kidney from the male *Cbr1^+/+^* and *Cbr1^+/-^* mice (n = 4 mice/group). (F) Quantification of CBR1 in the kidney from the male *Cbr1^+/+^* and *Cbr1^+/-^* mice. Data are expressed as mean ± SEM. Statistical analysis was conducted with the Mann-Whitney U test. ^∗^p < 0.05, ^∗∗^p < 0.01, and ^∗∗∗^p < 0.001.

**Figure S3**

**
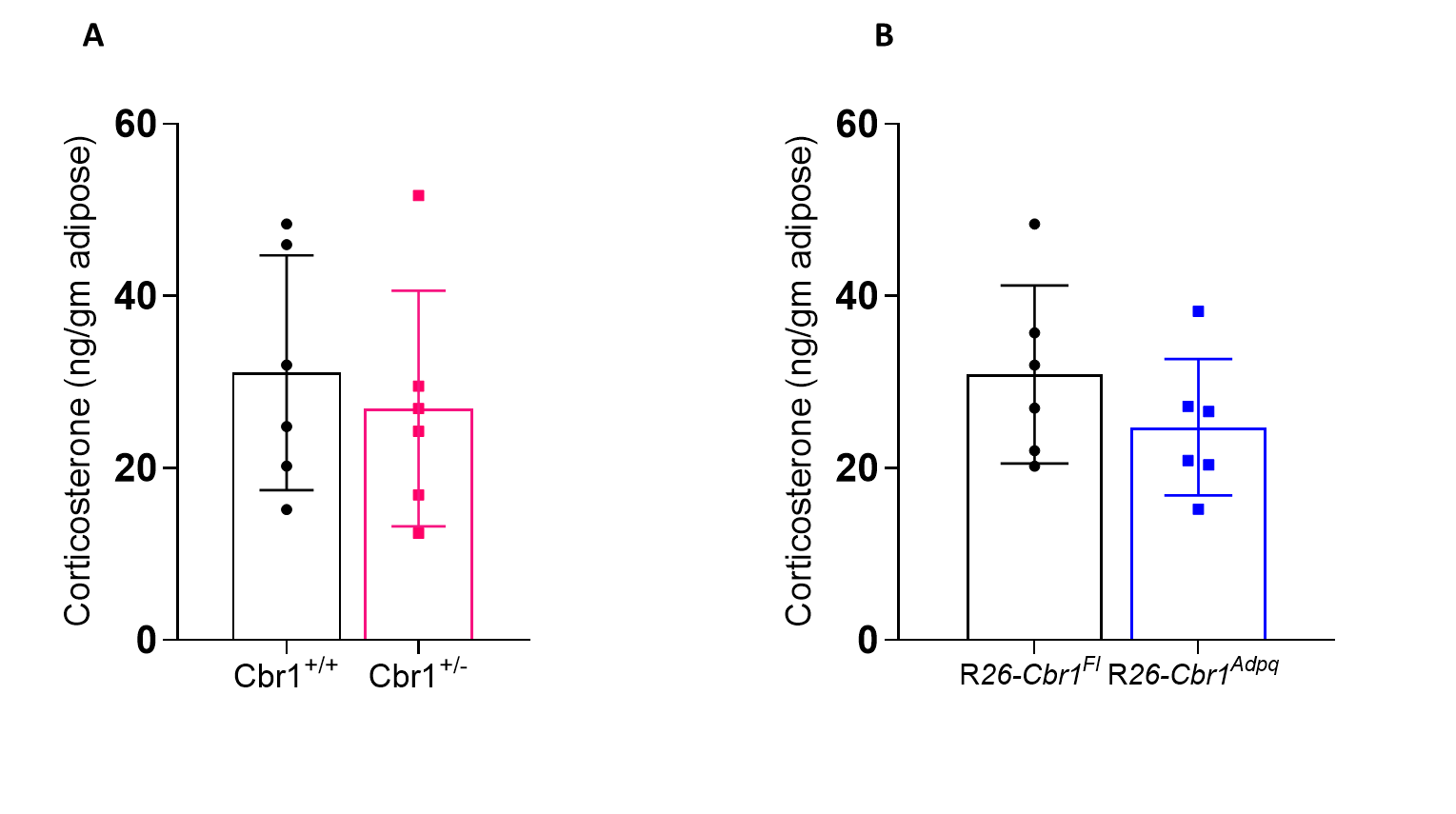
**

**Figure S3: Adipose corticosterone content was not different between the genotypes in either transgenic line.**

**Figure S4**

**
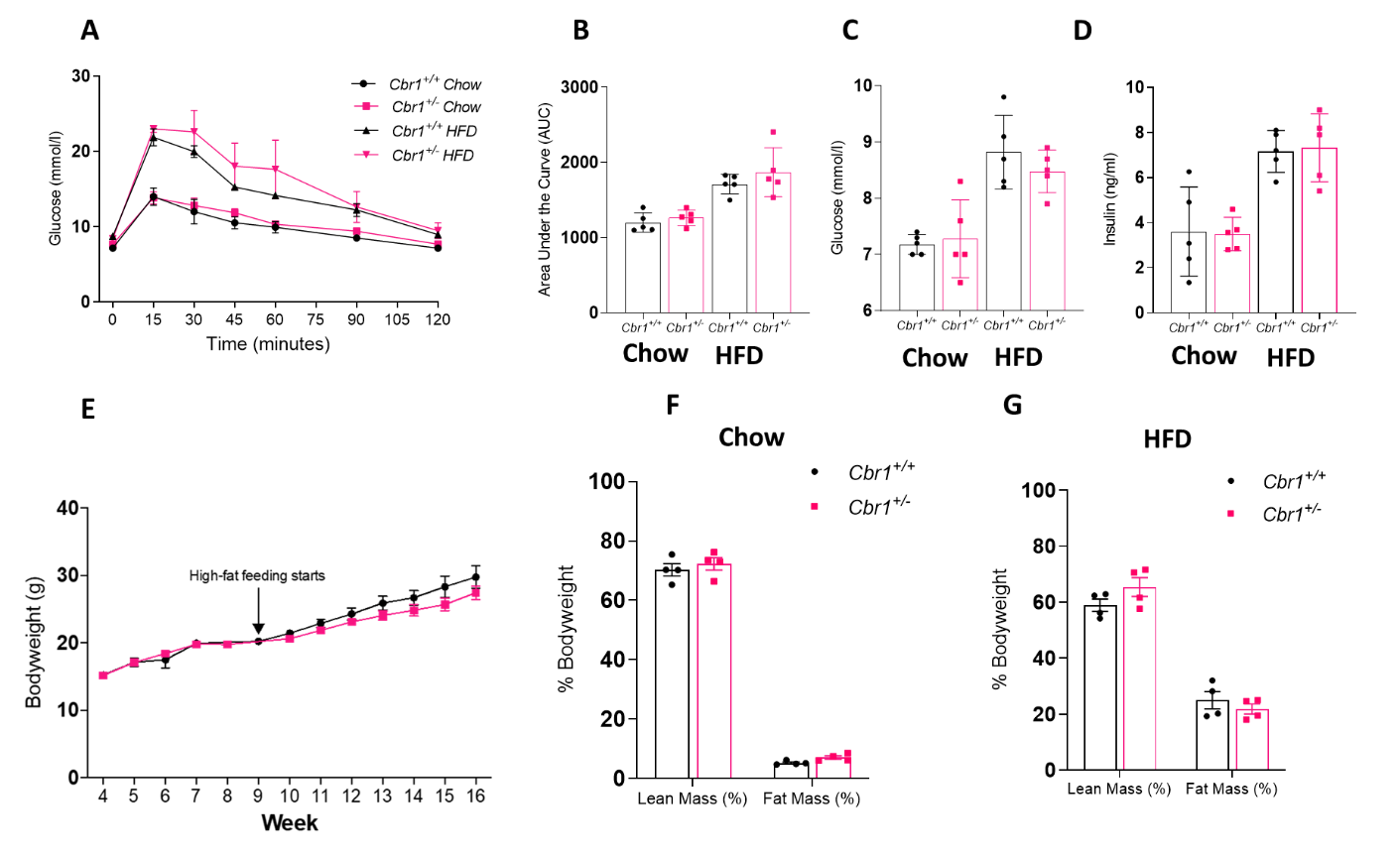
**

**Figure S4: Deletion of *Cbr1* did not alter glucose tolerance in female mice.** (A) Glucose tolerance tests (GTT) in the *Cbr1^+/+^* and *Cbr1^+/-^* mice (n = 5 mice/group) on the chow or high-fat diet. (B) Area under the curve for GTT. (C-D) Fasting plasma glucose and insulin concentrations in the *Cbr1^+/+^* and *Cbr1^+/-^* mice on the chow or high-fat diet (n = 5 mice/group). (E) Bodyweight gain with high-fat feeding in the *Cbr1^+/+^* and *Cbr1^+/-^* mice. (F) Lean mass and fat mass as a percentage of bodyweight in the *Cbr1^+/+^* and *Cbr1^+/-^* mice on the chow diet measured by TD-NMR (n = 5 mice/group). (G) Lean mass and fat mass as a percentage of bodyweight in the *Cbr1^+/+^* and *Cbr1^+/-^* mice on the high-fat diet measured by TD-NMR (n = 5 mice/group). Data are expressed as mean ± SEM. Statistical analysis was conducted with the Mann-Whitney U test. ^∗^p < 0.05, ^∗∗^p < 0.01, and ^∗∗∗^p < 0.001.

**Figure S5**

**
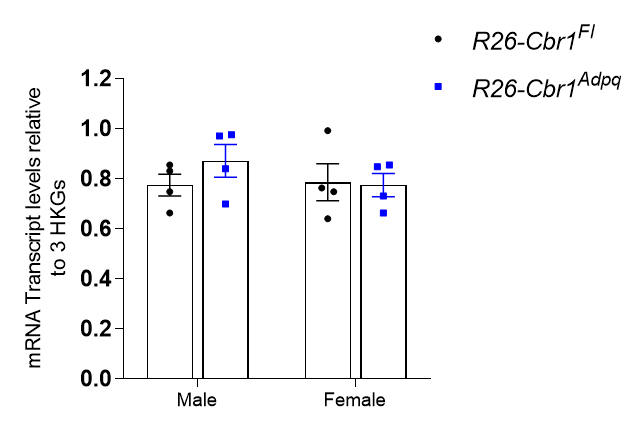
**

**Figure S5: Hepatic *Cbr1* mRNA expression was not different in the male and female *R26-Cbr1^Adpq^* mice compared with controls.**

**Figure S6**


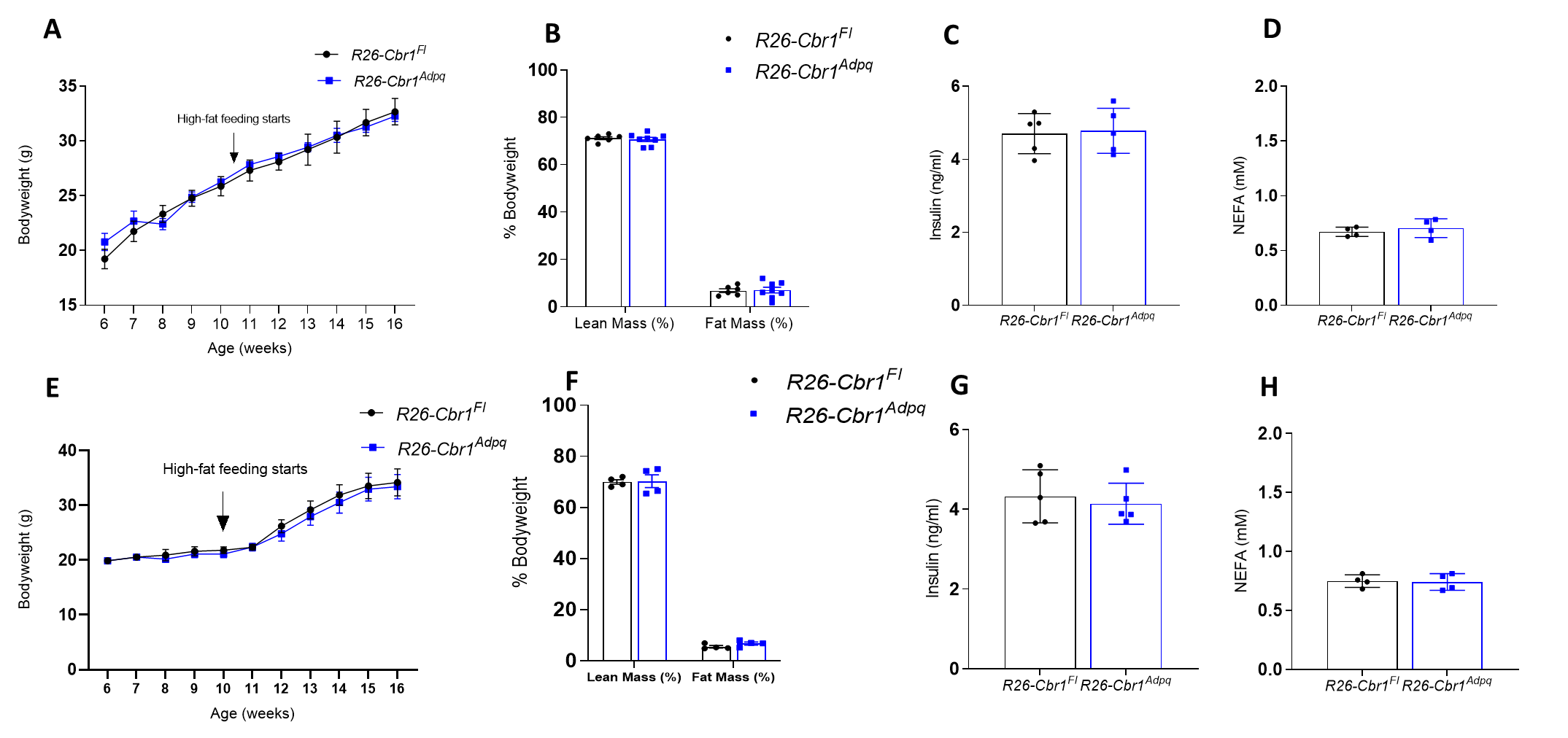


**Figure S6: Adipose-specific overexpression of Cbr1 did not affect bodyweight, insulin, or NEFAs on the chow diet in the males or females.** (A) Bodyweight over time in the male *R26-Cbr1^Fl^* and *R26-Cbr1^Adpq^* mice on the chow diet and after high-fat feeding (n = 5-7 mice/group). (B) Lean and fat mass percentage in the male *R26-Cbr1^Fl^* and *R26-Cbr1^Adpq^* mice on the chow diet. (C) Fasting plasma insulin concentrations in the male mice *R26-Cbr1^Fl^* and *R26-Cbr1^Adpq^* (n = 5 mice/group). (D) Fasting plasma non-esterified fatty acid concentrations in the male *R26-Cbr1^Fl^* and *R26-Cbr1^Adpq^* mice (n = 4 mice/group). (E) Bodyweight over time in the female *R26-Cbr1^Fl^* and *R26-Cbr1^Adpq^* mice on the chow diet and after high-fat feeding (n = 5-7 mice/group). (F) Lean and fat mass percentage in the female *R26-Cbr1^Fl^* and *R26-Cbr1^Adpq^* mice on the chow diet. (G) Fasting plasma insulin concentrations in the female *R26-Cbr1^Fl^* and *R26-Cbr1^Adpq^* mice (n = 5 mice/group). (H) Fasting plasma non-esterified fatty acid concentrations in the female *R26-Cbr1^Fl^* and *R26-Cbr1^Adpq^* mice (n = 4 mice/group). Data are mean ± SEM. Statistical significance was assessed by the Mann-Whitney U test and ANOVA indicating significance at ^*^p < 0.05 and ^**^p < 0.01.

**Supplementary tables**

**Supplementary Table 1. Primer details for qPCR**

| **Gene symbol, full name** | **Forward primer (3’ → 5’)** | **Reverse primer (5’ → 3’)** |
| --- | --- | --- |
| ***RNA18s, ribosomal RNA 18s*** | CTCAACACGGGAAACCTCAC | CGCTCCACCAACTAAGAACG |
| ***Tbp, TATA-binding protein*** | GGGAGAATCATGGACCAGAA | GATGGGAATTCCAGGAGTCA |
| ***β-actin*** | CTAAGGCCAACCGTGAAAAG | ACCAGAGGCATACAGGGACA |
| ***Cbr1, carbonyl reductase 1*** | AGGTGACAATGAAAACGAACTTT | GGACACATTCACCACTCTGC |
| ***mKate*** | GCACCCAGACCATGAGAATCAAG | CTGCCGTACATGAAGCTGGTA |
| ***GR*, *glucocorticoid receptor (*Nr3c1*)*** | CAAAGATTGCAGGTATCCTATGAA | CTTGGCTCTTCAGACCTTCC |
| ***MR*, m*ineralocorticoid receptor (*Nr3c2*)*** | CAAAAGAGCCGTGGAAGG | TTTCTCCGAATCTTATCAATAATGC |
| ***Pnpla2, adipose triglyceride lipase (Atgl)*** | GGTCCTTTGGTTCCACACAG | CCTCTCGAAGGCTCTCTTCC |
| ***Tsc22d3, glucocorticoid-induced leucine zipper protein (Gilz)*** | AGGTGGTTCTTCACGAGGTC | TCCGTTAAACTGGATAACAGTGC |
| ***Per1, period*** | GCTTCGTGGACTTGACACCT | TGCTTTAGATCGGCAGTGGT |
| ***Tnfα, tumour necrosis factor alpha*** | TGAGGAAGGCTGTGCATTG | GGCCTTCCTACCTTCAGACC |
| ***Ptdgs, prostaglandin D2 synthase*** | CTTCCAGCAGGACAAGTTCC | CGGGTCTCACACTGGTTTTT |

**Supplementary Table 2. Gene Ontology (GO) terms enriched in adipose exposed to 20β-dihydrocorticosterone compared with DMSO**

| **GO term** | **Genes** | **Number of significant genes** | **Number of genes in term** | **% of genes identified as significant within the term** | **Odds ratio, number of significant genes over that expected** | **Raw (unadjusted) p value from hypergeometric test** | **Adjusted p value (Benjamini and Hochberg, 1995)** |
| --- | --- | --- | --- | --- | --- | --- | --- |
| Mitochondrion | *1700021F05Rik, 2010107E04Rik, 2410015M20Rik, Abcb8, Acaa2, Acads, Acadvl, Acly, Aco2, Acsf2, Acsf3, Acsl3, Acsl5, Adcy10, Akr1b10, Atp5a1, Atp5b, Atp5d, Atp5e, Atp5g2, Atp5h, Atp5j2, Atp5k, Atp5o, Atpaf2, Bok, Cars2, Ccdc51, Ccdc58, Chchd10, Ciapin1, Cidea, Cisd3, Cmc2, Coasy, Coq6, Coq7, Cox10, Cox16, Cox4i1, Cox4i2, Cox5a, Cox6b1, Cox7a1, Cox7a2, Cox7c, Cox8b, Crym, Cs, Cyc1, Cycs, Dlat, Dlst, Dnajc11, Ecsit, Etfb, Gars, Gpd1, Gpd2, Grpel1, Hadha, Hccs, Hddc2, Hdhd3, Higd1a, Hk2, Kcnj8, Lace1, Lactb2, Lars2, Ldha, Ldhb, Letm1, Letmd1, Lipt1, Lrrk1, Mdh2, Minos1, Mpc1, Mpc2, Mrpl12, Mrpl16, Mrpl30, Mrpl34, Mrpl37, Mrpl51, Mrps10, Mrps27, Mrps28, Mrps35, Mtfp1, Mthfd2, Ndufa1, Ndufa11, Ndufa5, Ndufa9, Ndufb11, Ndufb5, Ndufb7, Ndufb8, Ndufs4, Ndufs5, Ndufs6, Ndufs8, Ndufv1, Nipsnap1, Pdhb, Pdp2, Pfdn4, Phb, Pkm, Pnkd, Ppif, Prkaca, Ptcd2, Raf1, Rhot2, Rilp, Rmdn3, Samm50, Sars2, Sdhb, Sdhd, Sept4, Sirt5, Slc25a19, Slc25a35, Sod2, Sox4, Suclg1, Tars2, Tbrg4, Timm17a, Timm44, Timm50, Tmem14c, Txnrd2, Ucp3, Uqcr10, Uqcr11, Uqcrc1, Uqcrh, Vars2, Vdac2, Xrcc3, Zadh2* | 146 | 1701 | 8.58 | 6.20 | 3.146e-52 | 9.012e-49 |
| Mitochondrial part | *2410015M20Rik, Abcb8, Acaa2, Acads, Acadvl, Acsl3, Acsl5, Atp5a1, Atp5b, Atp5d, Atp5e, Atp5g2, Atp5h, Atp5j2, Atp5k, Atp5o, Bok, Chchd10, Cidea, Coasy, Coq6, Coq7, Cox10, Cox16, Cox4i1, Cox4i2, Cox5a, Cox6b1, Cox7a1, Cox7a2, Cox7c, Cox8b, Cs, Cyc1, Cycs, Dlat, Dnajc11, Etfb, Gpd2, Grpel1, Hadha, Hccs, Higd1a, Hk2, Lactb2, Letm1, Letmd1, Mdh2, Minos1, Mpc1, Mpc2, Mrpl12, Mrpl16, Mrpl30, Mrpl34, Mrpl37, Mrpl51, Mrps10, Mrps28, Mrps35, Mtfp1, Ndufa1, Ndufa11, Ndufa5, Ndufa9, Ndufb11, Ndufb5, Ndufb7, Ndufb8, Ndufs4, Ndufs5, Ndufs6, Ndufs8, Ndufv1, Nipsnap1, Phb, Ppif, Rhot2, Rmdn3, Samm50, Sdhb, Sdhd, Sirt5, Slc25a19, Slc25a35, Sod2, Suclg1, Timm17a, Timm44, Timm50, Tmem14c, Ucp3, Uqcr10, Uqcr11, Uqcrc1, Uqcrh, Vdac2* | 97 | 739 | 13.13 | 8.80 | 3.155e-49 | 4.520e-46 |
| Mitochondrial inner membrane | *2410015M20Rik, Abcb8, Acaa2, Acadvl, Acsl5, Atp5a1, Atp5b, Atp5d, Atp5e, Atp5g2, Atp5h, Atp5j2, Atp5k, Atp5o, Coq6, Coq7, Cox4i1, Cox4i2, Cox5a, Cox6b1, Cox7a1, Cox7a2, Cox7c, Cox8b, Cyc1, Dnajc11, Gpd2, Grpel1, Hadha, Hccs, Higd1a, Letm1, Letmd1, Mdh2, Minos1, Mpc1, Mpc2, Mtfp1, Ndufa1, Ndufa11, Ndufa5, Ndufa9, Ndufb11, Ndufb5, Ndufb7, Ndufb8, Ndufs4, Ndufs5, Ndufs6, Ndufs8, Ndufv1, Nipsnap1, Phb, Ppif, Rhot2, Samm50, Sdhb, Sdhd, Sirt5, Slc25a19, Slc25a35, Sod2, Suclg1, Timm17a, Timm44, Timm50, Tmem14c, Ucp3, Uqcr10, Uqcr11, Uqcrc1, Uqcrh, Vdac2* | 73 | 398 | 18.34 | 12.33 | 6.128e-47 | 5.853e-44 |
| Organelle inner membrane | *2410015M20Rik, Abcb8, Acaa2, Acadvl, Acsl5, Atp5a1, Atp5b, Atp5d, Atp5e, Atp5g2, Atp5h, Atp5j2, Atp5k, Atp5o, Coq6, Coq7, Cox4i1, Cox4i2, Cox5a, Cox6b1, Cox7a1, Cox7a2, Cox7c, Cox8b, Cyc1, Dnajc11, Gpd2, Grpel1, Hadha, Hccs, Higd1a, Letm1, Letmd1, Mdh2, Minos1, Mpc1, Mpc2, Mtfp1, Ndufa1, Ndufa11, Ndufa5, Ndufa9, Ndufb11, Ndufb5, Ndufb7, Ndufb8, Ndufs4, Ndufs5, Ndufs6, Ndufs8, Ndufv1, Nipsnap1, P2rx5, Phb, Ppif, Rhot2, Samm50, Sdhb, Sdhd, Sirt5, Slc25a19, Slc25a35, Sod2, Suclg1, Timm17a, Timm44, Timm50, Tmem14c, Ucp3, Uqcr10, Uqcr11, Uqcrc1, Uqcrh, Vdac2* | 74 | 438 | 16.89 | 11.17 | 5.818e-45 | 4.167e-42 |
| Mitochondrial envelope | *2410015M20Rik, Abcb8, Acaa2, Acads, Acadvl, Acsl3, Acsl5, Atp5a1, Atp5b, Atp5d, Atp5e, Atp5g2, Atp5h, Atp5j2, Atp5k, Atp5o, Bok, Chchd10, Cidea, Coq6, Coq7, Cox10, Cox16, Cox4i1, Cox4i2, Cox5a, Cox6b1, Cox7a1, Cox7a2, Cox7c, Cox8b, Cyc1, Cycs, Dnajc11, Gpd2, Grpel1, Hadha, Hccs, Higd1a, Hk2, Letm1, Letmd1, Mdh2, Minos1, Mpc1, Mpc2, Mtfp1, Ndufa1, Ndufa11, Ndufa5, Ndufa9, Ndufb11, Ndufb5, Ndufb7, Ndufb8, Ndufs4, Ndufs5, Ndufs6, Ndufs8, Ndufv1, Nipsnap1, Phb, Ppif, Rhot2, Rmdn3, Samm50, Sdhb, Sdhd, Sirt5, Slc25a19, Slc25a35, Sod2, Suclg1, Timm17a, Timm44, Timm50, Tmem14c, Ucp3, Uqcr10, Uqcr11, Uqcrc1, Uqcrh, Vdac2* | 83 | 586 | 14.16 | 9.25 | 2.066e-44 | 1.184e-41 |

**Supplementary Table 3. Kyoto Encyclopaedia of Genes and Genomes (KEGG) terms enriched in adipose exposed to 20β-dihydrocorticosterone compared with DMSO**

| **KEGG term** | **Genes** | **Number of significant genes** | **Number of genes in term** | **% of genes identified as significant within the term** | **Odds ratio, number of significant genes over that expected** | **Raw (unadjusted) p value from hypergeometric test** | **Adjusted p value (Benjamini and Hochberg, 1995)** |
| --- | --- | --- | --- | --- | --- | --- | --- |
| Oxidative phosphorylation | Atp4b, Atp5a1, Atp5b, Atp5d, Atp5e, Atp5g2, Atp5h, Atp5j2, Atp5k, Atp5o, Cox10, Cox4i1, Cox4i2, Cox5a, Cox6b1, Cox7a1, Cox7a2, Cox7c, Cox8b, Cyc1, Ndufa1, Ndufa11, Ndufa5, Ndufa9, Ndufb11, Ndufb5, Ndufb7, Ndufb8, Ndufs4, Ndufs5, Ndufs6, Ndufs8, Ndufv1, Sdhb, Sdhd, Uqcr10, Uqcr11, Uqcrc1, Uqcrh | 39 | 133 | 29.32 | 18.32 | 1.642e-30 | 2.775e-28 |
| Parkinson's disease | Atp5a1, Atp5b, Atp5d, Atp5e, Atp5g2, Atp5h, Atp5o, Cox4i1, Cox4i2, Cox5a, Cox6b1, Cox7a1, Cox7a2, Cox7c, Cox8b, Cyc1, Cycs, Ndufa1, Ndufa11, Ndufa5, Ndufa9, Ndufb11, Ndufb5, Ndufb7, Ndufb8, Ndufs4, Ndufs5, Ndufs6, Ndufs8, Ndufv1, Ppif, Prkaca, Sdhb, Sdhd, Uqcr10, Uqcr11, Uqcrc1, Uqcrh, Vdac2 | 39 | 142 | 27.46 | 16.70 | 2.686e-29 | 2.269e-27 |
| Huntington's disease | Atp5a1, Atp5b, Atp5d, Atp5e, Atp5g2, Atp5h, Atp5o, Cox4i1, Cox4i2, Cox5a, Cox6b1, Cox7a1, Cox7a2, Cox7c, Cox8b, Cyc1, Cycs, Dnah7a, Ndufa1, Ndufa11, Ndufa5, Ndufa9, Ndufb11, Ndufb5, Ndufb7, Ndufb8, Ndufs4, Ndufs5, Ndufs6, Ndufs8, Ndufv1, Polr2g, Ppif, Sdhb, Sdhd, Sod2, Uqcr10, Uqcr11, Uqcrc1, Uqcrh, Vdac2 | 41 | 191 | 21.47 | 12.13 | 3.429e-26 | 1.932e-24 |
| Alzheimer's disease | Atp5a1, Atp5b, Atp5d, Atp5e, Atp5g2, Atp5h, Atp5o, Calm5, Cox4i1, Cox4i2, Cox5a, Cox6b1, Cox7a1, Cox7a2, Cox7c, Cox8b, Cyc1, Cycs, Ndufa1, Ndufa11, Ndufa5, Ndufa9, Ndufb11, Ndufb5, Ndufb7, Ndufb8, Ndufs4, Ndufs5, Ndufs6, Ndufs8, Ndufv1, Sdhb, Sdhd, Uqcr10, Uqcr11, Uqcrc1, Uqcrh | 37 | 174 | 21.26 | 11.69 | 1.771e-23 | 7.481e-22 |
| Non-alcoholic fatty liver disease (NAFLD) | Cox4i1, Cox4i2, Cox5a, Cox6b1, Cox7a1, Cox7a2, Cox7c, Cox8b, Cyc1, Cycs, Mlxipl, Ndufa1, Ndufa11, Ndufa5, Ndufa9, Ndufb11, Ndufb5, Ndufb7, Ndufb8, Ndufs4, Ndufs5, Ndufs6, Ndufs8, Ndufv1, Prkab1, Sdhb, Sdhd, Uqcr10, Uqcr11, Uqcrc1, Uqcrh | 31 | 149 | 20.81 | 10.97 | 1.960e-19 | 6.623e-18 |
